# MBD2 couples DNA methylation to transposable element silencing during male gametogenesis

**DOI:** 10.1038/s41477-023-01599-3

**Published:** 2024-01-15

**Authors:** Shuya Wang, Ming Wang, Lucia Ichino, Brandon A. Boone, Zhenhui Zhong, Ranjith K. Papareddy, Evan K. Lin, Jaewon Yun, Suhua Feng, Steven E. Jacobsen

**Affiliations:** 1 Molecular Biology Institute, University of California, Los Angeles, Los Angeles, CA USA; 2 Department of Molecular, Cell and Developmental Biology, University of California, Los Angeles, Los Angeles, CA USA; 3 State Key Laboratory of Integrated Management of Pest Insects and Rodents, Institute of Zoology, Chinese Academy of Sciences, Beijing, China; 4 Department of Chemical and Systems Biology, Stanford University School of Medicine, Stanford, CA USA; 5 Eli & Edythe Broad Center of Regenerative Medicine & Stem Cell Research, University of California, Los Angeles, Los Angeles, CA USA; 6 Howard Hughes Medical Institute, University of California, Los Angeles, Los Angeles, CA USA

**Keywords:** Gene silencing, Epigenomics, Plant molecular biology

## Abstract

DNA methylation is an essential component of transposable element (TE) silencing, yet the mechanism by which methylation causes transcriptional repression remains poorly understood^1–5^. Here we study the *Arabidopsis thaliana* Methyl-CpG Binding Domain (MBD) proteins MBD1, MBD2 and MBD4 and show that MBD2 acts as a TE repressor during male gametogenesis. MBD2 bound chromatin regions containing high levels of CG methylation, and MBD2 was capable of silencing the *FWA* gene when tethered to its promoter. MBD2 loss caused activation at a small subset of TEs in the vegetative cell of mature pollen without affecting DNA methylation levels, demonstrating that MBD2-mediated silencing acts strictly downstream of DNA methylation. TE activation in *mbd2* became more significant in the *mbd5* *mbd6* and *adcp1* mutant backgrounds, suggesting that MBD2 acts redundantly with other silencing pathways to repress TEs. Overall, our study identifies MBD2 as a methyl reader that acts downstream of DNA methylation to silence TEs during male gametogenesis.

## Abstract (web summary)

Transposable element proliferation poses considerable threats to genomes. A previously unknown methyl reader, MBD2, represses transposable elements during male gametogenesis. MBD2 acts with a high degree of redundancy with other silencing pathways, together preserving genome stability.

## Main

**Extended Data Fig. 1:**
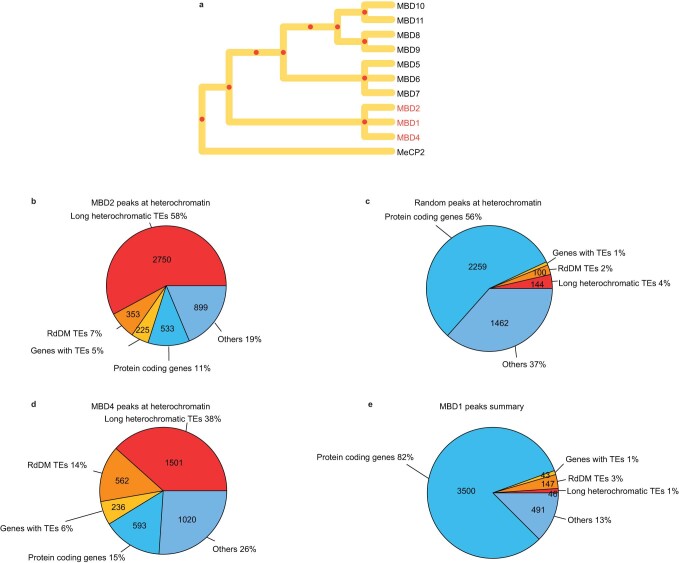
MBD2 and MBD4 are novel methyl readers. **a**. Phylogenetic tree of the MBD proteins, generated using the conserved sequences within the MBD domains by Clustal Omega (Supplementary Table 2). MeCP2 is used as the outgroup. Red dots indicate the hypothetical common ancestors. **b-e**. Proportions of heterochromatic peaks called from **b**. MBD2 ChIP-seq, **c**. random shuffling, **d**. MBD4 ChIP-seq, and peaks called from **e**. MBD1 ChIP-seq that overlap with long heterochromatic TEs, RdDM-associated TEs, genes with TEs, protein-coding genes, and others.

DNA methylation at transposable elements (TEs) usually causes transcriptional silencing, and the underlying mechanisms involve, in part, the recruitment of methyl reader proteins^1–4,6–11^. For example, in *Arabidopsis thaliana*, two functionally redundant Methyl-CpG Binding Domain (MBD) proteins, MBD5 and MBD6, bind methylated sites and prevent a subset of TEs from activation^8^. MBD1, MBD2 and MBD4 form a monophyletic group (Extended Data Fig. 1a), yet key evidence on their methyl-binding capacity and regulatory roles at TEs is lacking. Here we demonstrate that MBD2 and MBD4 bound DNA methylated chromatin regions, while MBD1 localized to unmethylated chromatin. MBD2 could silence *FWA* and other genes when tethered to these sites with an artificial zinc finger, suggesting a repressive role of this methyl reader. In addition, the loss of MBD2 caused TE activation in the vegetative cell during male gametogenesis without altering DNA methylation levels. This TE activation was enhanced when knocking out MBD5/MBD6 or ADCP1 in the *mbd2* mutant background. These results suggest that MBD2 is a methyl reader acting downstream of DNA methylation and collaborates with other silencing pathways to safeguard the genome from TE activity during male gametogenesis.

**Fig. 1:**
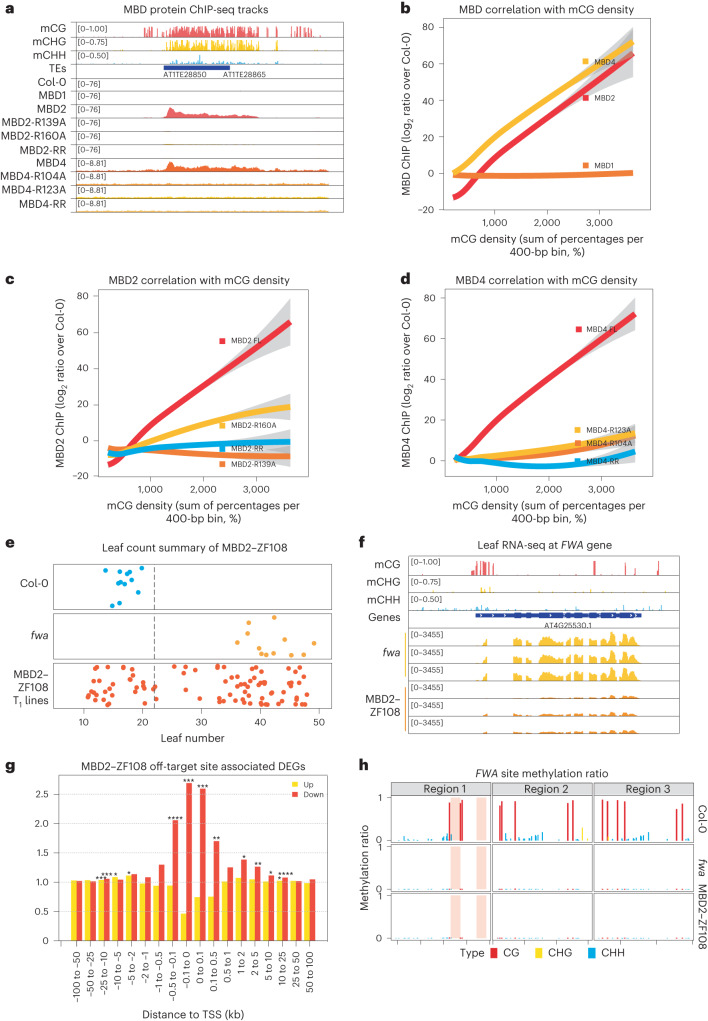
MBD2 is a methyl reader that silences *FWA* exogenously. **a**, Screenshot of the ChIP-seq tracks of the Col-0 control, MBD1, MBD2, MBD4 and the corresponding arginine mutants (normalized by RPGC), together with the wild-type DNA methylation percentage at this representative TE site. **b**–**d**, The correlation (calculated by loess regression) between CG methylation density and ChIP-seq signal of MBD1, MBD2 and MBD4 (**b**); MBD2 and its arginine mutants (**c**); and MBD4 and its arginine mutants (**d**). FL represents the full-length version of MBDs, while RR represents the double arginine mutant of MBDs. The grey areas represent the 95% confidence intervals calculated by s.e. **e**, Flowering time of Col-0, *fwa* and MBD2–ZF018 T_1_ lines as measured by the number of leaves. The dashed line indicates plants with 22 leaves or fewer. **f**, Screenshot of the leaf RNA-seq tracks of *fwa* and three representative T_2_ lines of MBD2–ZF108 (normalized by RPKM), with the wild-type DNA methylation percentage at the *FWA* locus as reference. **g**, The observed versus expected values of the upregulated (yellow) and downregulated (red) differentially expressed genes (DEGs) close to the ZF108 off-target sites, measured by Region Associated DEG analysis^26^. The asterisks represent *P* values from one-sided hypergeometric tests: *P < 0.05; **P < 0.01; ***P < 0.001; ****P < 0.0001. TSS, transcription start site. **h**, CG, CHG and CHH methylation measured by BS-PCR at the *FWA* promoter region of Col-0, *fwa* and a representative early flowering T_2_ plant from an early flowering T_1_ plant of MBD2–ZF108. The chromosome locations are indicated as follows: Region1 (chr4: 13038160-13038320 bp), Region2 (chr4: 13038350-13038500 bp), and Region3 (chr4: 13038500-13038700 bp).

**Extended Data Fig. 2:**
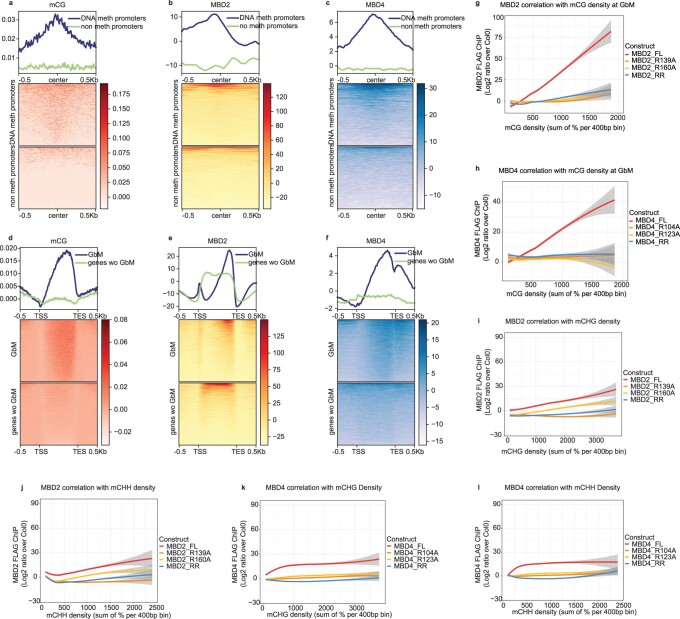
MBD2 and MBD4 enrich at DNA methylated promoters and gene bodies. **a-c**. Metaplots showing **a**. CG methylation, **b**. MBD2 ChIP-seq, and **c**. MBD4 ChIP-seq at DNA methylated promoters. **d-f**. Metaplots showing **d**. CG methylation, **e**. MBD2 ChIP-seq, and **f**. MBD4 ChIP-seq at genes with gene body methylation (GbM). **g-h**. The correlation between CG methylation density and **g**. MBD2 ChIP-seq or **h**. MBD4 ChIP-seq at GbM. **i-j**. The correlation between MBD2 ChIP-seq and **i**. CHG methylation density or **j**. CHH methylation density. **k-l**. The correlation between MBD4 ChIP-seq and **k**. CHG methylation density or **l**. CHH methylation density. FL represents the full-length version of MBDs while RR represents the double arginine mutant of MBDs. The grey area in Fig. 2g-l represents 95% confidence interval calculated by s.e.

MBD1, MBD2 and MBD4 all contain the conserved MBD domain^8,12–17^. MBD2 and MBD4 possess two conserved arginine residues predicted to form hydrogen-bond and π–cation interactions with methylated cytosines, while MBD1 contains only one of these arginines^8,12–17^. Previous studies have concluded that MBD1, MBD2 and MBD4 lack methyl-binding capacity from in vitro experiments^12–16,18^. However, it is possible that these in vitro experiments did not detect methyl binding because the MBD proteins lacked the required post-translational modifications and/or the buffer conditions were not ideal^19–21^. Since the genomic localizations of MBD1, MBD2 and MBD4 in vivo have not been determined, we generated transgenic lines expressing full-length MBD1, MBD2 and MBD4 driven by their endogenous promoters and performed chromatin immunoprecipitation sequencing (ChIP-seq). In line with the modelling results, MBD2 and MBD4, but not MBD1, displayed strong enrichment at highly methylated regions, including heterochromatic TE regions and TEs associated with RNA-directed DNA methylation (Fig. 1a, Extended Data Fig. 1b–e and Supplementary Table 1). MBD2 and MBD4 also displayed enrichment at DNA-methylated promoters, which typically contain RNA-directed-DNA-methylation-associated TEs (Extended Data Fig. 2a–c). In addition to TEs, MBD2 and MBD4 were enriched at the 3′ ends of genes with gene body methylation, following the CG methylation pattern (Extended Data Fig. 2d–f). Furthermore, MBD2 and MBD4 exhibited a positive correlation with CG methylation density at genes with gene body methylation and genome-wide, while the correlations with CHG and CHH methylation density were relatively weak (Fig. 1b and Extended Data Fig. 2g–l). This is consistent with MBD2 binding to oligonucleotides methylated in the CG context but not in the CHG and CHH contexts^22^. In contrast, MBD1 mainly localized to unmethylated genes and was not enriched at methylated loci (Fig. 1a,b and Extended Data Fig. 1e).

**Extended Data Fig. 3:**
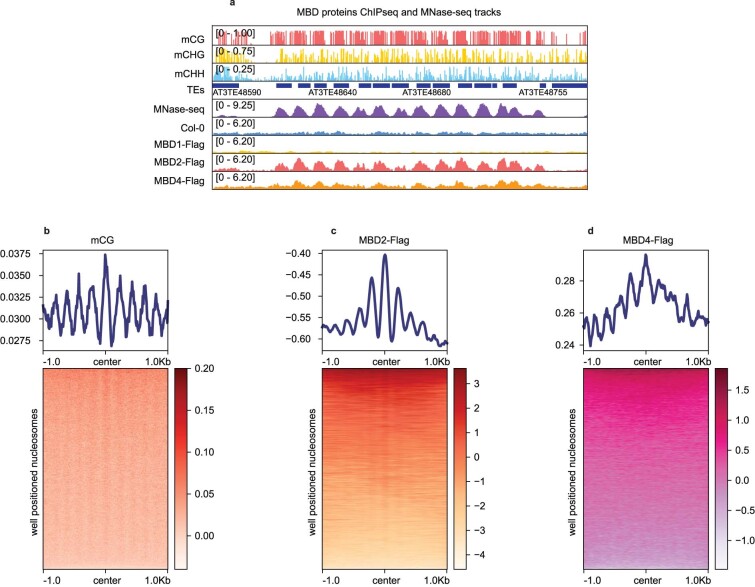
MBD2 and MBD4 follow DNA methylation at well positioned nucleosomes. **a**. Screenshot of the ChIP-seq tracks of MNase-seq, Col-0 control, MBD1, MBD2, and MBD4 (normalized by RPGC) with the wild-type DNA methylation percentage at the representative TE sites with well positioned nucleosomes. **b-d**. Metaplots and heat maps showing **b**. CG methylation, **c**. MBD2 ChIP-seq, or **d**. MBD4 ChIP-seq at well positioned nucleosomes.

To examine the necessity of the conserved arginines, we altered either or both arginines to alanine and assessed whether MBD2 and MBD4 lost their enrichment at methylated regions. Indeed, alterations of either arginine substantially diminished the methyl-binding capacity of MBD2 and MBD4, while the loss of both arginines completely abolished their preference for methylated chromatin (Fig. 1a,c,d). We also investigated the patterning of MBD2 and MBD4 at well-positioned nucleosomes within heterochromatin, since the deposition of CG methylation can be guided by nucleosomes at the replication fork^1–4^. MBD2 and MBD4 peaked at the centre of well-positioned nucleosomes, displaying a periodic binding pattern reflecting CG methylation density (Extended Data Fig. 3a–d). In summary, these results show that MBD2 and MBD4 bind methylated CG sites in the genome, in part using the conserved arginine residues thought to interact with methylated cytosines.

**Extended Data Fig. 4:**
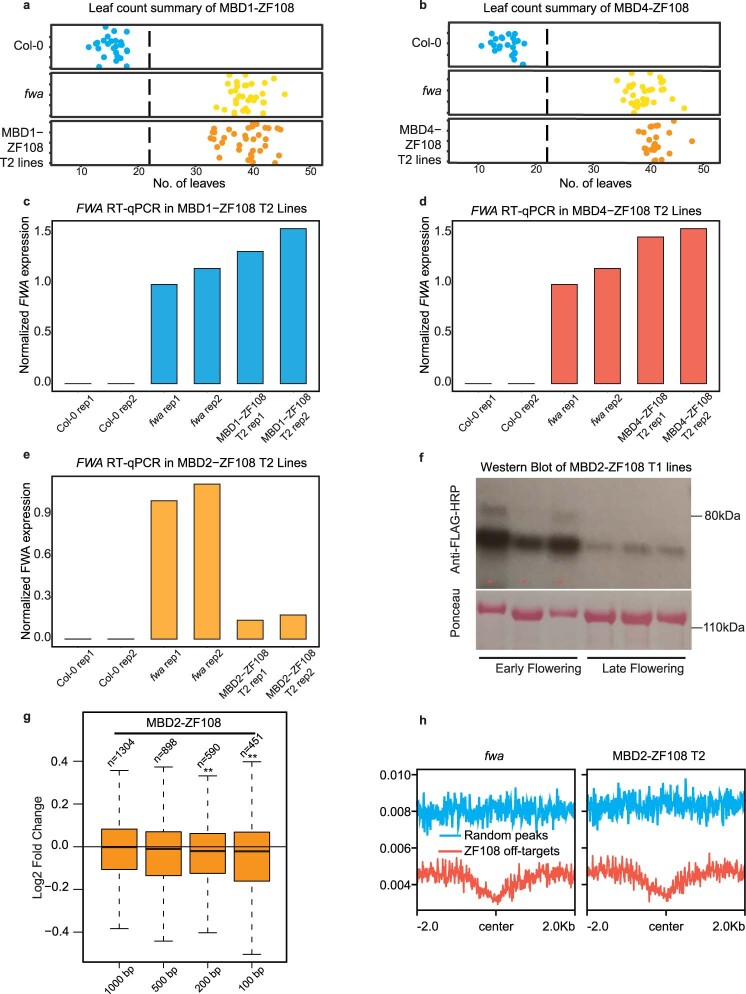
MBD2 but not MBD1 and MBD4 silences *FWA* exogenously. **a**, **b**. Flowering time of Col-0, *fwa*, and the representative T2 lines of **a**. MBD1-ZF108 and **b**. MBD4-ZF108 as measured by the number of leaves. **c-e**. qRT-PCR analysis showing the relative mRNA level of *FWA* in Col-0, *fwa*, and the representative T2 lines of **c**. MBD1-ZF108, **d**. MBD4-ZF108, and **e**. MBD2-ZF108. **f**. Western blot of the representative early flowering and late flowering T1 lines of MBD2-ZF108, which were used for flowering time measurement in Fig. 1e. Pink asterisk indicates early flowering T1 plants. **g**. Log_2_ fold change of the DEGs close to ZF108 off-target sites from MBD2-ZF108 leaf RNA-seq. The middle line in the box plots represents the median, the box shows the IQR, and the whiskers reach the minimum and maximum values. *n* represents the number of off-target sites. 3 biologically independent experiments were used. P value is calculated by two-sided parametric *t*-test, P < 0.01: ** **h**. CG methylation level at the ZF108 off-target sites and random sites of *fwa* and the representative T2 line of MBD2-ZF108. Source data

We tested whether MBD1, MBD2 or MBD4 could mediate transcriptional silencing when ectopically tethered to a gene promoter. Each MBD protein was fused with an artificial zinc finger domain (ZF108) designed to bind to the target gene *FWA*^23^. This gene encodes a transcription factor normally DNA methylated at its promoter and silent in vegetative tissues. However, *Arabidopsis* *fwa* epigenetic alleles have permanently lost the promoter DNA methylation, resulting in overexpression of *FWA* and a late-flowering phenotype in which plants produce an increased number of leaves prior to flowering^24^. When the MBD–ZF108 fusions were transformed into the *fwa* background, the MBD2–ZF108 fusion, but not the MBD1–ZF108 or MBD4–ZF108 fusion, effectively silenced *FWA* and caused an early flowering phenotype (Fig. 1e,f and Extended Data Fig. 4a–e). The wide distribution of flowering times in the MBD2–ZF108 T_1_ lines (Fig. 1e) is probably due to variable fusion protein expression levels, since three of the sampled early flowering plants showed high levels of expression as measured by western blots, while three sampled late-flowering plants showed very low protein levels (Extended Data Fig. 4f).

ZF108 is known to bind not only the *FWA* gene but also many off-target sites in the genome^25^. We performed Region Associated DEG analysis^26^ at off-target sites and found that MBD2–ZF108 efficiently silences genes whose promoters are close to the ZF108 off-target site (Fig. 1g and Extended Data Fig. 4g). To test whether MBD2–ZF108-induced gene silencing was associated with changes in DNA methylation, we performed bisulfite sequencing PCR (BS-PCR) and whole-genome bisulfite sequencing (WGBS). MBD2–ZF108 did not alter the methylation status of the *FWA* promoter or of the other ZF108 binding sites, indicating that MBD2-mediated silencing is independent of DNA methylation (Fig. 1h and Extended Data Fig. 4h). These results show that MBD2 can act as a silencing factor when tethered to promoters.

**Fig. 2:**
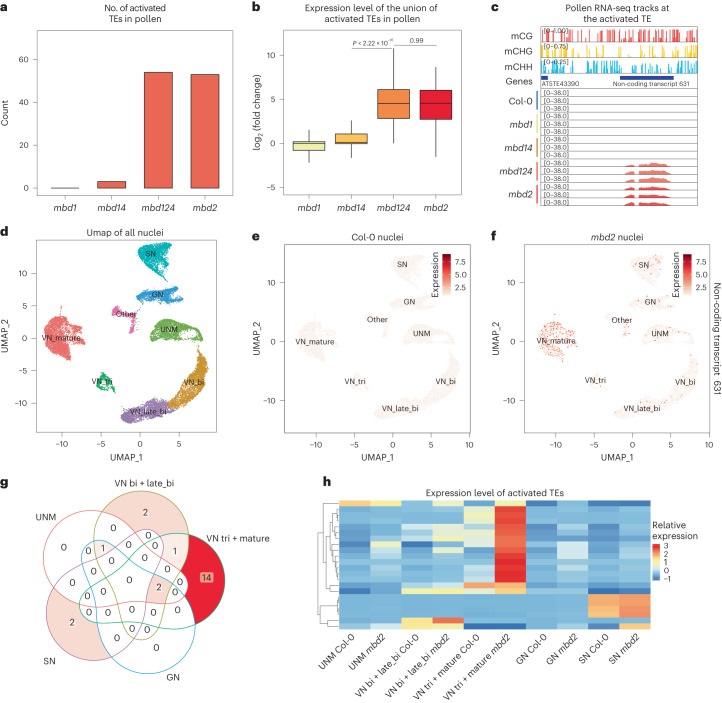
MBD2 silences TEs during male gametogenesis. **a**, Count of the activated TEs from mature pollen RNA-seq of *mbd1*, *mbd14*, *mbd124* and *mbd2* mutants. **b**, Log_2_ fold change of the activated TEs in *mbd1*, *mbd14*, *mbd124* and *mbd2* mutants. *n* = 71 TEs examined over three biologically independent experiments. The middle line in each box plot represents the median, the box shows the interquartile range (IQR) and the whiskers reach the minimum and maximum values. *P* values calculated by two-sided parametric *t*-tests are indicated. **c**, Screenshot of mature pollen RNA-seq tracks of Col-0, *mbd1*, *mbd14*, *mbd124* and *mbd2* (normalized by RPGC) with the wild-type DNA methylation percentage at the representative TE and the DNA methylated non-coding transcript (chr5: 12208690–12209360 bp). **d**, Uniform Manifold Approximation and Projection (UMAP) of the integrated Col-0 and *mbd2* snRNA-seq data with cluster annotations. UMN, microspores; GN, generative nuclei; SN, sperm nuclei; VN, vegetative nuclei; VN_bi, vegetative nuclei from bicellular pollen; VN_late_bi, vegetative nuclei from late bicellular pollen; VN_tri, vegetative nuclei from tricellular pollen. **e**,**f**, UMAPs of Col-0 and *mbd2* snRNA-seq showing the expression level of the representative DNA methylated transcript across clusters. **g**, Venn diagram showing the overlap of the activated TEs among UMAP clusters. **h**, Heat map showing the expression of the activated TE across clusters of Col-0 and *mbd2* snRNA-seq. The expression level is the scaled cluster averages.

**Extended Data Fig. 5:**
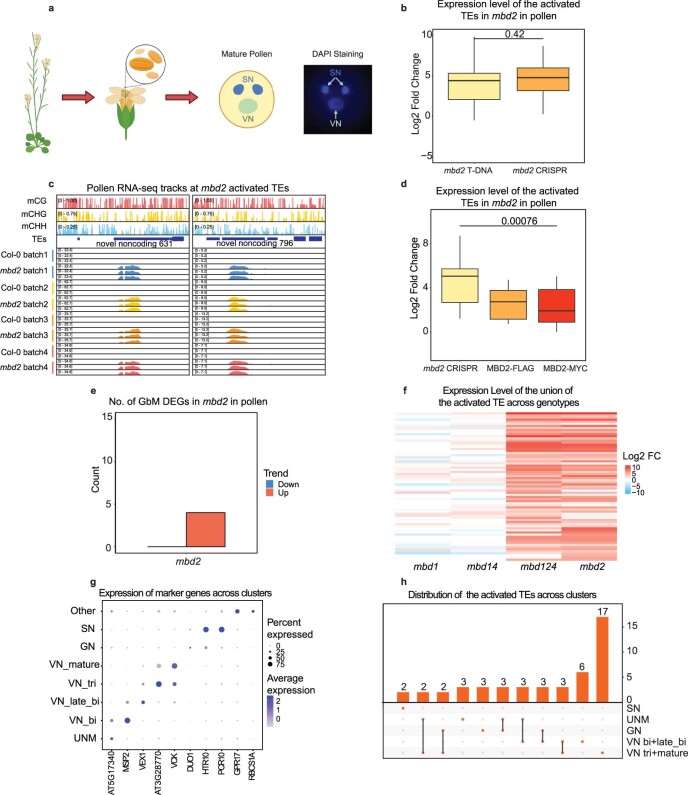
MBD2 prevents TE activation during the late stage of male gametogenesis. **a**. Graphic illustration showing the morphology and DAPI staining of wild-type mature pollen. **b**. Log_2_ fold change of the activated TEs in *mbd2* transfer DNA and CRISPR mutants. **c**. Screenshots of 4 batches of mature pollen RNA-seq tracks of Col-0 wild type and *mbd2* CRISPR mutant (normalized by RPGC) with wild-type DNA methylation percentage at the representative TEs. **d**. Log_2_ fold change of the activated TEs in *mbd2* CRISPR mutant and the complemented lines. **e**. Count of the differentially expressed transcripts at GbM from mature pollen RNA-seq of *mbd2* CRIPSR mutant. **f**. Heat map showing the expression level of activated TEs in *mbd1*, *mbd14*, *mbd124*, and *mbd2* mutants. **g**. Dot plot showing the cluster specificity using the expression of known markers. The dot size represents the percentage of cells in which the gene was detected. The dot color corresponds to the scaled average expression. **h**. Upset plot showing the distribution of activated TEs in the *mbd2* mutant across different clusters. In Fig. 5b, d, the middle line of the box plots represents the median, the box shows the IQR, and the whiskers reach the minimum and maximum values; *n* = 53 TEs over 3 biologically independent experiments; P values calculated by two-sided parametric *t*-test are indicated.

Two recent studies reported that an *mbd1* *mbd2* *mbd4* (*mbd124*) triple mutant showed upregulation at genes involved in biotic and abiotic stress in seedlings^27,28^, but no derepression was observed at methylated TEs where methyl readers typically bind and silence^27,28^. Because these previous studies were done using vegetative tissues and because MBD5 and MBD6 have been shown to prevent TE activation specifically in pollen cells^29^, we hypothesized that MBD2 might play a similar role in pollen cells. The vegetative nucleus of pollen (VN) is known to display decompacted heterochromatin due to reduced CG methylation, H1 and dimethylated H3 lysine 9 (H3K9me2)^3,4,29–33^ (Extended Data Fig. 5a), and this partially compromised silencing creates a sensitized background in which further loss of silencing factors can cause more significant TE derepression^29^. To examine whether the loss of MBD1, MBD2 and MBD4 induces transcriptional activation in pollen and to determine potential synergistic functions among the MBDs, we generated *mbd1*, *mbd14*, *mbd2* and *mbd124* mutants and performed RNA-seq using mature pollen. We found that both CRISPR and transfer DNA mutants of *mbd2* showed significant reactivation of around 50 TEs (Fig. 2a and Extended Data Fig. 5b), which was consistently observed in four independent rounds of mature pollen RNA-seq (Extended Data Fig. 5c). In addition, TE upregulation was rescued by re-introducing FLAG- and MYC-tagged MBD2 into the CRISPR mutant, demonstrating a direct role of MBD2 in repressing TEs (Extended Data Fig. 5d). While MBD2 was enriched at genes with gene body methylation, the loss of MBD2 barely caused transcriptional changes at these regions (Extended Data Fig. 5e). Consistent with the ZF108 fusion experiment, only the *mbd2* and *mbd124* mutations, but not *mbd1* or *mbd14*, triggered TE expression (Fig. 2a–c and Extended Data Fig. 5f). Furthermore, *mbd2* and *mbd124* induced a comparable level of derepression at the same TE sites (Fig. 2a–c and Extended Data Fig. 5f). These results indicate that it is only MBD2 that plays a role in preventing TE activation in mature pollen.

**Extended Data Fig. 6:**
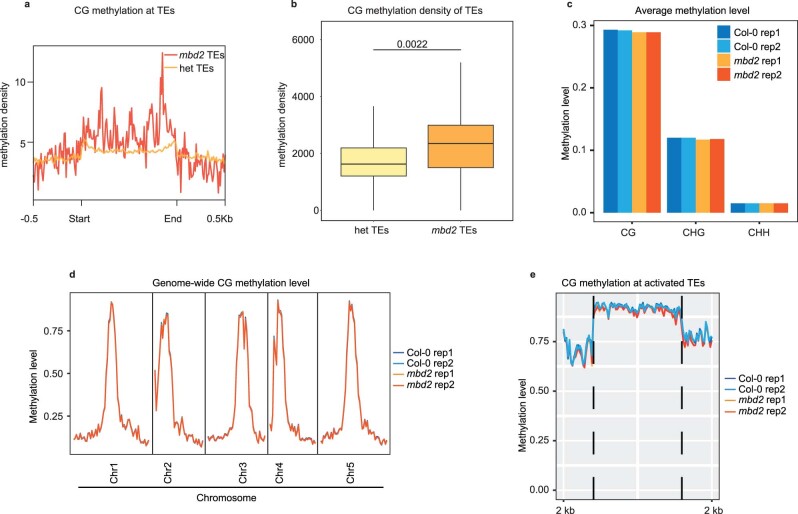
MBD2 silences TEs downstream of DNA methylation. **a**, **b**. CG methylation at the *mbd2* activated TEs (*n* = 53 TEs) and the heterochromatic TEs (*n* = 2094 TEs) shown by **a**. metaplot and **b**. boxplot. The middle line in the box plots represents the median, the box shows the IQR, and the whiskers reach the minimum and maximum values. 3 biologically independent experiments were used. P values calculated by two-sided parametric *t*-test are indicated. **c-d**. Genome-wide CG, CHG, and CHH methylation levels of Col-0 and *mbd2* mature pollen. **e**. CG methylation level at the *mbd2* activated TEs of Col-0 and *mbd2*.

To stage MBD2-mediated TE silencing during gametogenesis, we employed single-nucleus RNA-seq (snRNA-seq) in wild-type Columbia-0 (Col-0) and *mbd2*. This approach allowed us to distinguish between different nuclei types involved in male gametogenesis, including microspores, generative nuclei, sperm nuclei and VN^29^ (Fig. 2d and Extended Data Fig. 5g). We observed prominent derepression of DNA-methylated TEs in tricellular and mature VN nuclei in the *mbd2* mutant (Fig. 2e–h and Extended Data Figs. 5h and 6a,b). Interestingly, this pattern was different from that previously seen in *mbd5* *mbd6* (*mbd56*) mutants, in which derepression was more prominent in the early stages of VN development^29^. These different methyl readers thus coordinate stage-specific TE repression to safeguard the genome during the maturation of male gametophytes.

To test whether the loss of MBD2 affects DNA methylation, we compared the methylation levels of mature pollen of Col-0 and that of the *mbd2* mutant using WGBS. We detected no genome-wide changes in DNA methylation levels, suggesting that the loss of MBD2 does not impact global DNA methylation (Extended Data Fig. 6c,d). We also examined the TE sites that are activated in *mbd2* and again found no changes in DNA methylation levels (Extended Data Fig. 6e). These results indicate that MBD2 functions as a methyl reader that acts strictly downstream of DNA methylation and is not involved in the maintenance of DNA methylation patterns.

**Fig. 3:**
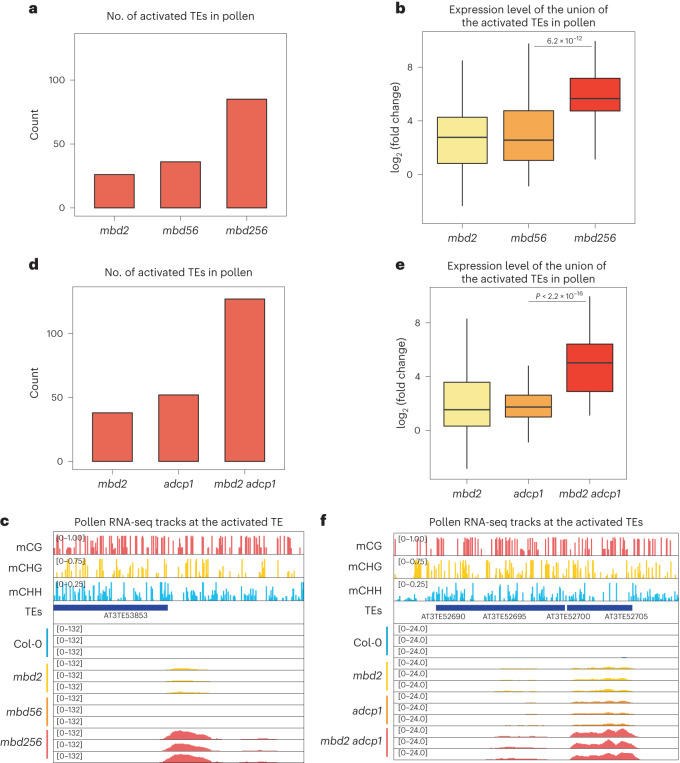
MBD2 prevents TE activation with other redundant pathways. **a**, Count of the activated TEs from mature pollen RNA-seq of *mbd2*, *mbd56* and *mbd256*. **b**, Log_2_ fold change of the activated TEs in *mbd2*, *mbd56* and *mbd256*. *n* = 86 TEs. **c**, Screenshots of mature pollen RNA-seq tracks of Col-0, *mbd2*, *mbd56* and *mbd256* (normalized by RPGC) with the wild-type DNA methylation percentage at the representative TE. **d**, Count of the activated TEs from mature pollen RNA-seq of *mbd2*, *adcp1* and *mbd2* *adcp1*. **e**, Log_2_ fold change of the activated TEs in *mbd2*, *adcp1* and *mbd2* *adcp1*. *n* = 129 TEs. **f**, Screenshots of mature pollen RNA-seq tracks of Col-0, *mbd2*, *adcp1* and *mbd2* *adcp1* (normalized by RPGC) with the wild-type DNA methylation percentage at the representative TEs. In **b** and **e**, the middle line in each box plot represents the median, the box shows the IQR and the whiskers reach the minimum and maximum values. Three biologically independent experiments were used; *P* values calculated by two-sided parametric *t*-tests are indicated.

**Extended Data Fig. 7:**
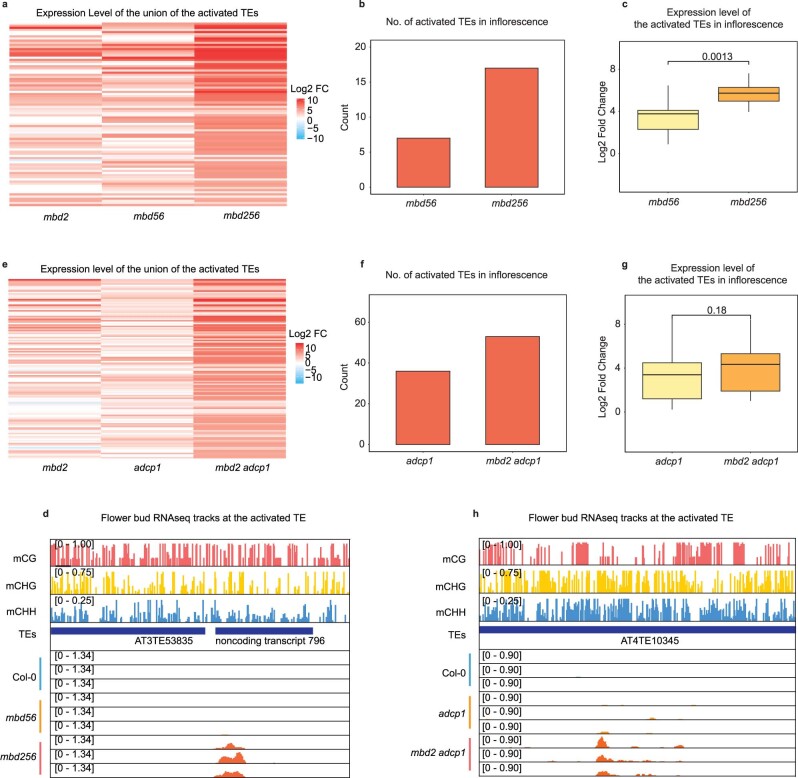
MBD2 silences TE redundantly with MBD5/6 and ADCP1. **a**. Heat map showing the expression of the activated TEs in *mbd2*, *mbd56*, and *mbd256* from mature pollen RNA-seq. **b**. Count of the activated TEs of *mbd56* and *mbd256* from inflorescence RNA-seq. **c**. Log_2_ fold change of the activated TEs of *mbd56* and *mbd256* from inflorescence RNA-seq. *n* = 17 TEs. **d**. Screenshot of the inflorescence RNA-seq tracks of Col-0, *mbd56*, and *mbd256* (normalized by RPGC) with the wild-type DNA methylation percentage at the representative TE and the DNA methylated transcript. **e**. Heat map showing the expression of the activated TEs in *mbd2*, *adcp1*, and *mbd2 adcp1* from mature pollen RNA-seq. **f**. Count of the activated TEs of *adcp1* and *mbd2 adcp1* from inflorescence RNA-seq. **g**. Log_2_ fold change of the activated TEs of *adcp1* and *mbd2 adcp1* from inflorescence RNA-seq. *n* = 51 TEs. **h**. Screenshot of the inflorescence RNA-seq tracks of Col-0, *adcp1*, and *mbd2 adcp1* (normalized by RPGC) with the wild-type DNA methylation percentage at the representative TE. In Fig. 7c, g, the middle line of the box plots represents the median, the box shows the IQR, and the whiskers reach the minimum and maximum values; 3 biologically independent experiments were used; P values calculated by two-sided parametric *t*-test are indicated.

To further study possible redundancies of MBD2 silencing with other pathways, we combined *mbd2* with *mbd56* since both MBD2 and MBD5/6 prevent TE activation during male gametogenesis. We performed mature pollen RNA-seq experiments comparing an *mbd2* *mbd5* *mbd6* (*mbd256)* triple mutant with *mbd2* and *mbd56* mutants. The *mbd256* triple mutant showed derepression of around 90 TEs, a higher number than in either *mbd2* or *mbd56* (Fig. 3a). Furthermore, when we examined the union of TEs activated in all mutants, *mbd256* displayed a roughly eightfold higher activation relative to *mbd2* and *mbd56* (Fig. 3b,c and Extended Data Fig. 7a). Such an enhancement was also observed in inflorescence tissues (Extended Data Fig. 7b–d), which is probably because they contain pollen. These data suggest that MBD2 silences TEs redundantly with MBD5/6.

We also considered that MBD2-mediated silencing might act redundantly with silencing pathways related to the epigenetic mark H3K9me2, a hallmark of *Arabidopsis* heterochromatin. A previous study reported that Agenet Domain Containing Protein 1 (ADCP1), an H3K9me2 reader, maintains silencing and the integrity of the heterochromatin compartment via a mechanism related to liquid–liquid phase separation^34^. We therefore generated an *mbd2* *adcp1* double mutant using CRISPR–Cas9 and performed mature pollen RNA-seq of Col-0, *mbd2*, *adcp1* and *mbd2* *adcp1* mutants. We observed a dramatic enhancement of TE activation in the *mbd2* *adcp1* double mutant compared with the *mbd2* and *adcp1* single mutants (Fig. 3d–f and Extended Data Fig. 7e). The *mbd2* *adcp1* double mutant had TE derepression at ~100 more sites than the single mutants, which had around 50 TEs activated, and also exhibited a much higher degree of derepression at the co-activated TEs (Fig. 3d–f and Extended Data Fig. 7e). Moreover, we found that *mbd2* *adcp1* showed activation in more TEs than did *adcp1* in inflorescence tissues, though to a lesser extent than mature pollen (Extended Data Fig. 7f–h). These findings suggest that MBD2 and ADCP1 redundantly suppress a group of TEs, and either component is sufficient to keep these regions silent.

**Fig. 4:**
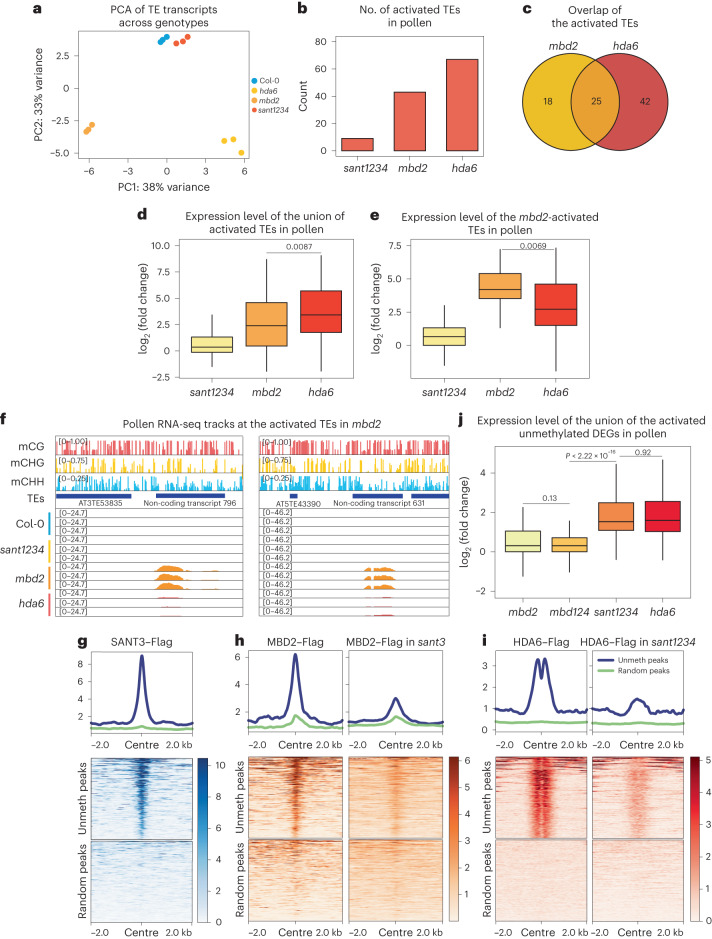
MBD2-mediated TE silencing is independent of HDA6 and SANT family proteins. **a**, PCA of Col-0, *mbd2*, *sant1234* and *hda6* based on the TE transcripts from mature pollen RNA-seq. **b**, Count of the activated TEs from mature pollen RNA-seq of *mbd2*, *sant1234* and *hda6*. **c**, Overlap of the activated TEs in *mbd2* and *hda6*. **d**,**e**, Log_2_ fold change of the union of the activated TEs (*n* = 85 TEs) (**d**) and *mbd2*-activated TEs (*n* = 43 TEs) (**e**) in *mbd2*, *sant1234* and *hda6*, normalized to Col-0. **f**, Screenshots of mature pollen RNA-seq tracks of Col-0, *mbd2*, *sant1234* and *hda6* (normalized by RPGC) with the wild-type DNA methylation percentage at the representative TEs and DNA-methylated non-coding transcripts. **g**–**i**, Metaplots and heat maps showing the ChIP-seq signals of SANT3, MBD2, MBD2 in *sant3*, HDA6 and HDA6 in *sant1234* at the shared unmethylated (unmeth) peaks and random peaks. **j**, Log_2_ fold change of the activated unmethylated DEGs in *mbd2*, *mbd124*, *sant1234* and *hda6*, normalized to Col-0. *n* = 96 DEGs. In **d**, **e** and **j**, the middle line in each box plot represents the median, the box shows the IQR and the whiskers reach the minimum and maximum values. Three biologically independent experiments were used; *P* values calculated by two-sided parametric *t*-tests are indicated.

**Extended Data Fig. 8:**
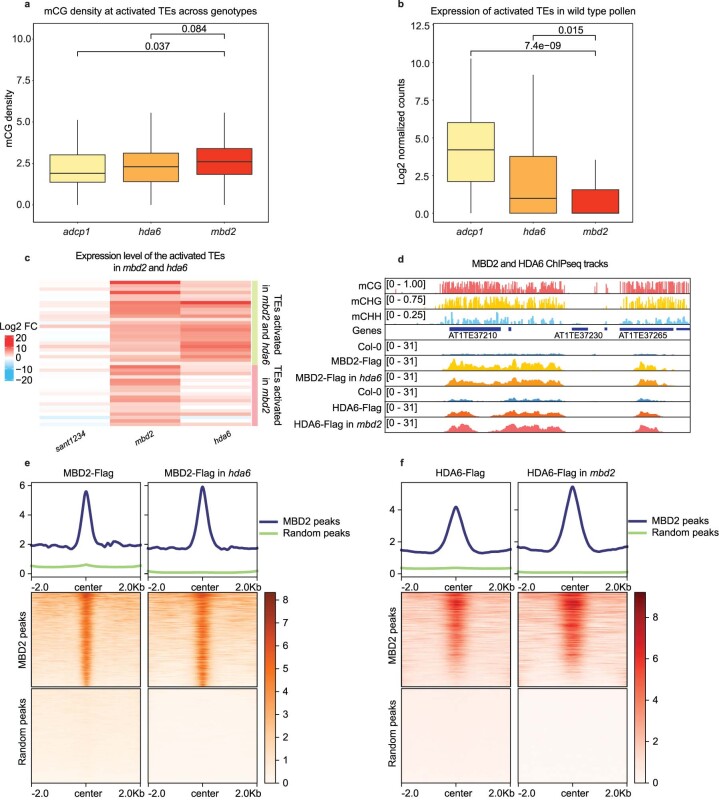
MBD2 mediated silencing is independent of HDA6 and SANT family proteins. **a**, **b**. Boxplots showing **a**. CG methylation density and **b**. basal expression level from mature pollen of *adcp1* (*n* = 52 TEs), *hda6* (*n* = 85 TEs), and *mbd2* (*n* = 53 TEs) activated TEs. The middle line in the box plots represents the median, the box shows the interquartile range (IQR), and the whiskers reach the minimum and maximum values. 3 biologically independent experiments were used. P values calculated by two-sided parametric *t*-test are indicated. **c**. Heat map showing the log_2_ fold change of the activated TEs in *sant1234*, *mbd2*, and *hda6* from mature pollen RNA-seq. **d**. Screenshot of the ChIP-seq tracks of Col-0 control, MBD2, MBD2 in *hda6*, HDA6, and HDA6 in *mbd2* (normalized by RPGC) with the wild-type DNA methylation percentage at the representative TEs. **e-f**. Metaplots and heat maps showing the ChIP-seq of **e**. MBD2, MBD2 in *hda6*, **f**. HDA6, and HDA6 in *mbd2* at the DNA methylated MBD2 peaks shared with HDA6 and random peaks.

Previous work has suggested that HDA6 and SANT3 cooperate with MBD1, MBD2 and MBD4 to regulate protein-coding gene expression involved in stress and flowering control in seedlings, and affinity purification–mass spectrometry has demonstrated interactions between MBD2, SANT3 and HDA6 (refs. ^27,28^). This led us to test whether a similar mechanism could underlie MBD2-mediated TE silencing in the VN. To dissect the individual and collective roles of these proteins in TE silencing, we performed mature pollen RNA-seq on *mbd2*, *hda6* and a *sant1* *sant2* *sant3* *sant4* quadruple mutant (*sant1234*)^27^. Principal component analysis (PCA) showed that the *sant1234* samples clustered closely with the Col-0 controls, suggesting that the SANT family is not involved in heterochromatin TE silencing (Fig. 4a). Furthermore, the *mbd2* and *hda6* samples diverged from Col-0 but formed two distinct clusters (Fig. 4a), suggesting that *mbd2* and *hda6* probably induce TE activation differently. The differences in the degree of TE activation and in the TEs that MBD2 and HDA6 repress probably account for the divergence between *mbd2* and *hda6* in the PCA (Fig. 4a). A higher proportion of *mbd2*-activated TEs belong to the LTR/Gypsy family, which are predominantly within deep heterochromatin. HDA6 silences a greater proportion of LTR/Copia TEs than MBD2, and these TEs are less commonly found in deep heterochromatin regions (Supplementary Table 2). In addition, *mbd2*-activated TEs are of higher CG methylation density and lower basal expression than *hda6*-activated TEs (Extended Data Fig. 8a,b). While the number of derepressed TEs in *sant1234* was very small, *mbd2* and *hda6* mutants displayed activation at more than 40 TEs (Fig. 4b). Among the activated TEs in *mbd2*, only around 50% were also activated in *hda6* (Fig. 4c). In addition, although *hda6* led to a slightly higher degree of upregulation at the union of the activated TEs (Fig. 4d and Extended Data Fig. 8c), we found that within the group of TEs activated in *mbd2*, TE transcript levels were nearly eight times higher in *mbd2* than in *hda6* (Fig. 4e,f and Extended Data Fig. 8c). These data suggest that, while HDA6 is clearly important in TE silencing in pollen, HDA6 is probably not the primary mechanism through which MBD2 represses TEs.

To further examine the relationship between MBD2 and HDA6, we generated transgenic lines expressing flag-tagged HDA6 or MBD2 in either Col-0 or mutant backgrounds and performed ChIP-seq using inflorescence tissues. At genomic regions that showed ChIP-seq signals for both HDA6 and MBD2 in Col-0 and that are also DNA methylated, we observed that HDA6 ChIP-seq signals were not diminished in the *mbd2* mutant and that MBD2 ChIP-seq signals were not diminished in the *hda6* mutant (Extended Data Fig. 8d–f). These results suggest that MBD2 does not recruit HDA6 to its targeted regions, or vice versa. These ChIP-seq data are consistent with the RNA-seq results and suggest that HDA6 is probably not responsible for MBD2-mediated TE repression.

**Extended Data Fig. 9:**
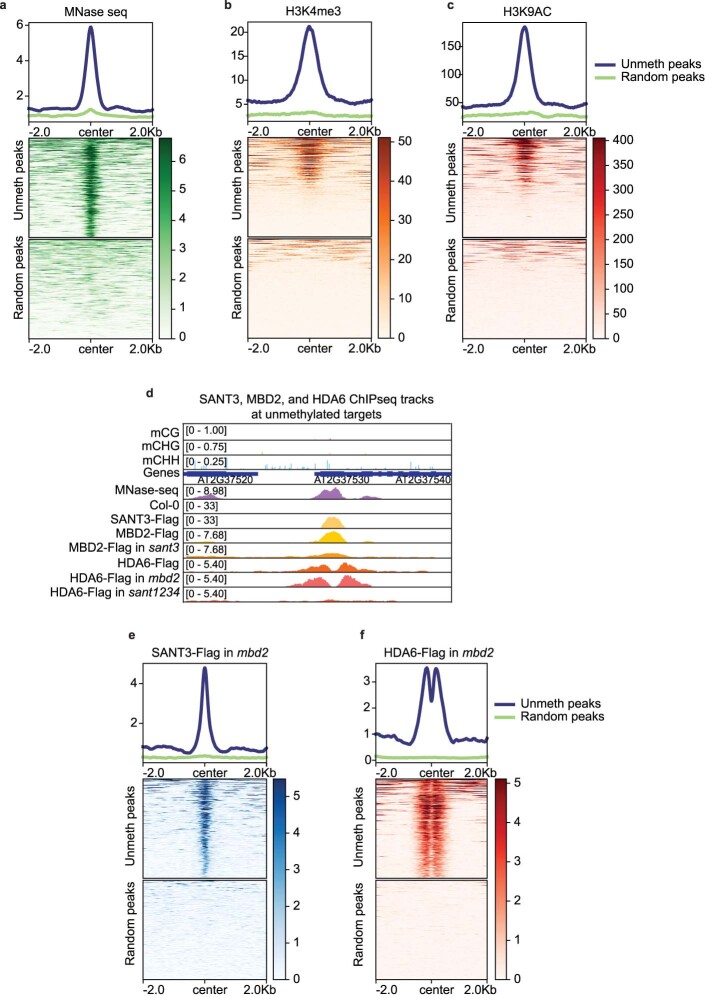
MBD2 forms a complex with HDA6 and SANT family proteins at +1 nucleosomes of unmethylated genes. **a-c**. Metaplots and heat maps showing **a**. MNase-seq, **b**. H3K4me3, and **c**. H3K9ac enrichment at the unmethylated peaks and random peaks. **d**. Screenshot of the ChIP-seq tracks of Col-0 control and the indicated samples (normalized by RPGC) with the wild-type DNA methylation percentage at the representative unmethylated genes. **e-f**. Metaplots and heat maps showing the ChIP-seq signal of **e**. SANT3 in *mbd2*, and **f**. HDA6 in *mbd2* at the unmethylated peaks and random peaks.

**Extended Data Fig. 10:**
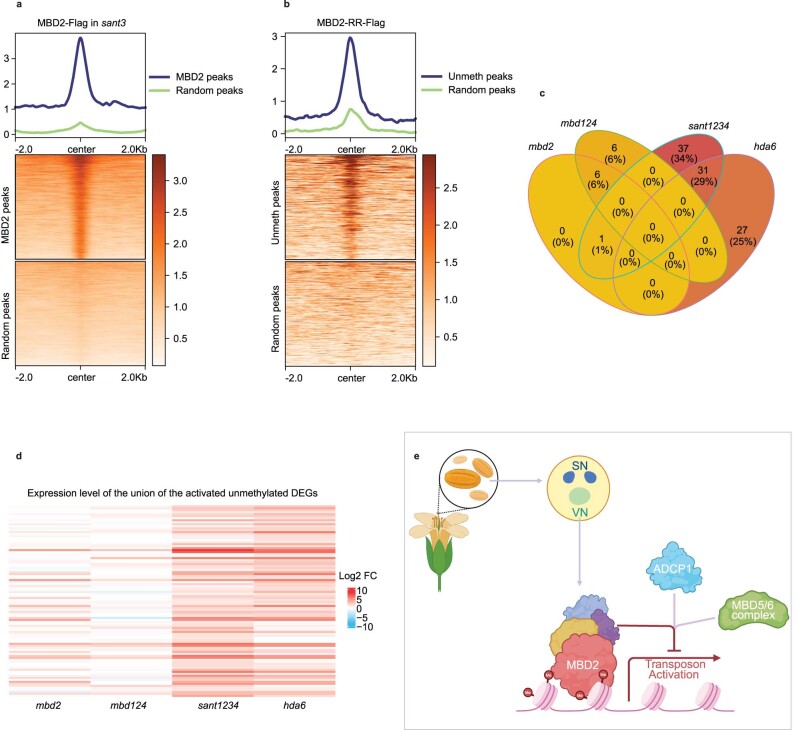
Distinct mechanisms mediate MBD2 behavior at unmethylated and methylated regions. Metaplots and heat maps showing the ChIP-seq signal of **a**. MBD2 in *sant3* at the DNA methylated MBD2 peaks shared with HDA6 and random peaks, and **b**. MBD2-RR at unmethylated peaks shared among MBD2, SANT3, and HDA6, and random peaks. **c**. Venn diagram showing the overlap of activated unmethylated DEGs among *mbd2*, *mbd124*, *sant1234*, and *hda6*. **d**. Heat map showing the expression level of unmethylated DEGs in *mbd2*, *mbd124*, *sant1234*, and *hda6* from mature pollen RNA-seq. **e**. Graphic representation summarizing the main function of MBD2 during male gametogenesis.

In light of the involvement of SANT family proteins and HDA6 in unmethylated gene regulation in seedlings^27,28^, we sought to dissect the formation of the SANT/HDA6/MBD2 complex and further explore its biological functions. Using ChIP-seq, we identified around 1,000 euchromatic peaks that did not overlap with TEs, did not contain DNA methylation and were shared among SANT3, MBD2 and HDA6 (Fig. 4g–i). Interestingly, these peaks were primarily found at the +1 nucleosome of moderately transcribed genes marked by active histone modifications, including H3K4me3 and H3K9ac (Extended Data Fig. 9a–d). Additionally, HDA6 displayed a dual and symmetric peak pattern around the +1 nucleosome at these sites, which is consistent with recent structural insights on dimerized yeast HDACs bound at the two sides of a mononucleosome to access H3/H4 and H2B tails simultaneously^35^ (Fig. 4i). We found that the ChIP-seq signals of SANT3 and HDA6 in the *mbd2* mutant were unaltered at the ~1,000 shared regions, demonstrating that MBD2 is dispensable for the localization of SANT3 and HDA6 (Extended Data Fig. 9e,f). However, in the absence of SANT3, MBD2 exhibited a dramatic reduction at these peaks (Fig. 4h). Additionally, since HDA6 interacts with all SANT family proteins^27,28^, we examined HDA6 enrichment at these peaks in the *sant1234* mutant and found that most of the HDA6 signal was lost at these sites (Fig. 4i). Collectively, these ChIP-seq results suggest that SANT3, potentially along with other SANT proteins, recruits MBD2 and HDA6 to unmethylated genes. In contrast, the *sant3* mutation had no effect on the localization of MBD2 at its methylated target sites (Extended Data Fig. 10a). Moreover, while the MBD2 double arginine mutation caused a loss of binding to DNA-methylated regions, it had no effect on the localization of MBD2 to its unmethylated targets (Extended Data Fig. 10b). These results demonstrate that MBD2 recruitment to unmethylated and methylated sites operates by different mechanisms: MBD2 binding to unmethylated sites requires SANT3, while binding to methylated sites does not involve SANT3 but requires critical amino acids in the methyl-binding domain.

Since MBD2 regulates TEs in pollen, we sought to test whether MBD2, together with SANTs and HDA6, might also act to repress unmethylated genes in pollen. Revisiting the mature pollen RNA-seq of Col-0 and the *mbd2*, *sant1234* and *hda6* mutants, we found that while *mbd2* showed activation of only a few unmethylated genes, *sant1234* and *hda6* showed upregulation of a much larger set (Fig. 4j and Extended Data Fig. 10c). Furthermore, while there was a large overlap in the upregulated genes found in *sant1234* and *hda6*, there was no overlap between these genes and the genes upregulated in *mbd2* (Extended Data Fig. 10c). Considering the potential redundancy with MBD1 and MBD4, we also reanalysed the mature pollen RNA-seq of *mbd124* and again found only a few upregulated genes, and these showed no overlap with the upregulated genes in *sant1234* and *hda6* (Fig. 4j and Extended Data Fig. 10c,d). These results suggest that MBD2 plays only a minor role at unmethylated genes in pollen, while SANTs and HDA6 play a much more prominent and MBD2-independent role. This is consistent with previous work showing that the *mbd124* triple mutant had a much weaker activation of protein-coding genes than the *sant1234* and *hda6* mutants in seedling tissues^27,28^. Overall, these results indicate that, while MBD2 is localized to some unmethylated genes and interacts with SANT and HDA6 proteins that are also present at these genes, MBD2 has little function at these sites and mainly functions as a repressor of DNA-methylated TEs.

In summary, this work demonstrates that MBD2 functions as a methyl reader that maintains TE silencing in pollen, while its close homologues MBD1 and MBD4 play no such role. Even though MBD4 does bind to methylated chromatin and is expressed in mature pollen (Supplementary Table 3), it does not have a TE-silencing phenotype in pollen. It seems possible that MBD4 plays a repressive role in other specific tissues or functions redundantly with unknown silencing pathways. It is also possible that MBD4 is an evolutionary remnant of a methylated-DNA-binding protein that has lost its silencing capacity. MBD2 silences TEs downstream of DNA methylation through a mechanism that does not require the SANT or HDA6 proteins. MBD2-mediated silencing is also distinct from the MBD5/6 and ACDP1 silencing pathways. These results highlight a high degree of redundancy between different silencing pathways acting downstream of DNA methylation, each contributing to the critical and immense function of maintaining repression of different types of TEs (Extended Data Fig. 10e). This multitude of silencing pathways probably reflects the intense evolutionary competition between TE proliferation and the plant genomes’ response to silence TEs and preserve genome integrity.

## Methods

### Phylogenetic analysis

Highly conserved MBD domain sequences of MBD1, MBD2, MBD4, MBD5, MBD6, MBD7, MBD8, MBD9, MBD10, MBD11 and human MeCP2 were taken for phylogenetic analysis. All the sequences are listed in Supplementary Table 4. Protein sequence alignments were performed using Clustal Omega^36–38^. Graphic representation of the phylogenetic tree was generated using iTOL (v.6.7.5)^39^. Human MeCP2 was used as an outgroup given its evolutionary distance from *Arabidopsis* MBDs.

### Plant materials and growth conditions

The plants used in this paper were *Arabidopsis thaliana* Col-0 ecotype and were grown under long-day conditions (16 h light and 8 h dark). Seedlings of Col-0 and the *mbd2* CRISPR mutant were harvested after ten days of incubation under long-day conditions. The transfer DNA insertion lines used in this study are *mbd1* (SALK_025352), *mbd2* (GABI_650A05), *mbd4* (SALK_042834), *mbd6* (SALK_043927), *hda6* (SALK_201895C) and *sant3* (SALK_004966). The CRISPR mutants were generated using the pYAO::hSpCas9 system^40^. The *mbd2* CRISPR mutant was generated using the guides ACCGTAAATGCCCCGATAGA and CTAGGTACGCCAACCGAGTC. The *mbd5* CRISPR mutant was generated using the guides TCACGGAAACGTGCGACGCC and ACTTAGTATTTACTGATCGT. The *adcp1* CRISPR mutant was generated using the same guides as in Zhao et al.^34^: ATTCCGCGGCTCGTGGTACATGG and GGCAGCTACCACTGAAAGGAGGG. The *sant1234* mutant is from a previous study^27^. Detailed information on high-order mutants generated in this study is summarized in Supplementary Table 5. Transgenic plants were generated through floral dipping using *Agrobacterium* (AGL0 strain).

### Plasmid construction

The Gateway-compatible binary destination vector, pEG302–effector (gDNA)–3xFLAG, was used to generate FLAG-tagged proteins for the ChIP-seq experiments. The plasmid contains a Gateway cassette, a carboxy-terminal 3xFLAG epitope tag, a Biotin Ligase Recognition Peptide and an OCS terminator. Genomic sequences starting from the native promoter (the sequence includes ~1.5 kb upstream from the 5′ untranslated region or the intergenic sequences before the 5′ untranslated region) to the end of the endogenous gene (without the stop codon) were cloned into pENTR D-TOPO vectors (Invitrogen), from which the genomic sequences were switched into the destination vector using Gateway LR Clonase II (Invitrogen). To generate constructs for the ZF108 targeting experiments, pEG302–effector (gDNA)–3xFLAG–ZF108, another Gateway-compatible binary destination vector, was used. The plasmid contains a Gateway cassette, a C-terminal 3xFLAG epitope tag, a ZF108 motif, a Biotin Ligase Recognition Peptide and an OCS terminator. The cloning strategy was the same as for pEG302–effector (gDNA)–3xFLAG. pMDC123–UBQ10–effector (cDNA) ZF108–3xFLAG was also used for the ZF108 targeting experiments. The plasmid is a Gateway-compatible binary destination vector that consists of a plant UBQ10 promoter, a C-terminal ZF108 motif, a 3xFLAG peptide, a Gateway cassette and an OCS terminator. The cDNA was first cloned into pENTR D-TOPO vectors (Invitrogen) and then translocated into the PMDC123 destination vector via the LR reaction using Gateway LR Clonase II (Invitrogen). pYAO::hSpCas9 plasmid was used to generate the CRISPR mutants. The guides of MBD2, MBD5, MBD6 and ADCP1 were amplified via overlapping PCR (primer tails containing the guide sequence) using AtU6-26-sgRNA cassette as the template. Purified PCR products were cloned into pYAO::hSpCas9 plasmid via In-Fusion (Takara, 639650).

### Quantitative PCR with reverse transcription

Rosette leaf tissues from three- to four-week-old plants were collected. RNA was extracted using the Zymo Direct-zol RNA MiniPrep kit (Zymo Research). Between 400 ng and 1 μg of total RNA was used for reverse transcription with Superscript III First Strand Synthesis Supermix (Invitrogen). Finally, quantitative PCR was performed with iQ SYBR Green Supermix (Bio-Rad), and *FWA* expression was normalized to ISOPENTENYL PYROPHOSPHATE DIMETHYLALLYL PYROPHOSPHATE ISOMERASE 2 (IPP2). The primers are listed here:

**Table:** 

Primer name	Primer sequence
*FWA* forward	TTAGATCCAAAGGAGTATCAAAG
*FWA* reverse	CTTTGGTACCAGCGGAGA
IPP2 forward	GTATGAGTTGCTTCTCCAGCAAAG
IPP2 reverse	GAGGATGGCTGCAACAAGTGT

### ChIP-seq

For ChIP-seq, all buffers, if not specified, were supplemented with PMSF (Sigma), benzamidine (Sigma) and cOmpleteTM Protease Inhibitor Cocktail (Sigma). In short, 1–2 g of unopened flower buds or 2–4 g of rosette leaves from the T_2_ lines were collected and ground with liquid nitrogen. We used 25 ml of nuclei isolation buffer (50 mM HEPES, 1 M sucrose, 5 mM KCl, 5 mM MgCl_2_, 0.6% Triton X-100) to dissolve the nuclei and then added 680 μl of 37% formaldehyde to reach 1% formaldehyde concentration. The incubation lasted for 12 min before we added freshly made 2 M glycine solution to quench the crosslinking. After the purification using extraction buffer 2 (0.25 M sucrose, 10 mM Tris-HCl pH 8, 10 mM MgCl2, 1% Triton X-100, 5 mM BME) and extraction buffer 3 (1.7 M sucrose, 10 mM Tris-HCl pH 8, 2 mM MgCl_2_, 0.15% Triton X-100, 5 mM BME), the nuclei were lysed using nuclei lysis buffer (50 mM Tris pH 8, 10 mM EDTA, 1% SDS). The chromatin was sheared via Bioruptor Plus (Diagenode) (the settings were 30 s on/30 s off, high, repeat 22 cycles). Each sample was added to 1.7 ml of ChIP Dilution Buffer (1.1% Triton X-100, 1.2 mM EDTA, 16.7 mM Tris pH 8, 167 mM NaCl) and immunoprecipitated with 10 μl of anti-FLAG antibody (1:800 dilution, Sigma) overnight at 4 °C. The next day, 50 μl of Protein A and 50 μl of Protein G Dynabeads (Invitrogen) were combined, washed with ChIP Dilution Buffer and added to each sample. The incubation lasted for 2 h at 4 °C. Five rounds of washes were then applied to reduce the background: the samples were washed twice with Low Salt Buffer (150 mM NaCl, 0.2% SDS, 0.5% Triton X-100, 2 mM EDTA, 20 mM Tris pH 8), once with High Salt Buffer (200 mM NaCl, 0.2% SDS, 0.5% Triton X-100, 2 mM EDTA, 20 mM Tris pH 8), once with LiCl Buffer (250 mM LiCl, 1% Igepal, 1% sodium deoxycholate, 1 mM EDTA, 10 mM Tris pH 8) and once with TE buffer (10 mM Tris pH 8, 1 mM EDTA). Elution was done at 65 °C using 250 μl of elution buffer (1% SDS, 10 mM EDTA, 0.1 M NaHCO_3_) twice. We used 20 μl of the elution for western blot to check the pull-down of FLAG-tagged proteins with anti-FLAG M2-Peroxidase (HRP) antibody (1:10,000 dilution, Sigma). After elution, the DNA–protein complex was reverse-crosslinked using 20 μl of 5 M NaCl at 65 °C overnight. The next day, 1 μl of Proteinase K (Invitrogen), 10 μl of 0.5 M EDTA (Invitrogen) and 20 μl of 1 M Tris pH 6.5 were added to digest the proteins. The DNA was then purified using phenol:chloroform:isoamyl alcohol (Invitrogen) and precipitated with sodium acetate (Invitrogen), GlycoBlue (Invitrogen) and ethanol overnight at −20 °C. The next day, the precipitated DNA was collected and processed for the library using the Ovation Ultra Low System V2 kit (NuGEN). The sequencing was performed on an Illumina NovaSeq 6000.

### MNase-seq

The buffers for MNase-seq were supplemented with PMSF (Sigma), benzamidine (Sigma) and cOmpleteTM Protease Inhibitor Cocktail (Sigma). Around 0.5 g of Col-0 floral tissues were harvested fresh without flash-freezing. Then, 25 ml of nuclei isolation buffer (300 mM sucrose, 20 mM Tris-HCl pH 8, 5 mM MgCl_2_, 5 mM KCl, 0.2% Triton X-100, 5 mM BME, 35% glycerol, 1 mM EDTA) was added, and the samples were homogenized using a homogenizer (Omni International GLH) with the following settings: 1 min at level 2, 45 s at level 3 and 45 s at level 4. The samples were then passed through two-layer Miracloth and spun down for 20 min at 2,880 *g* at 4 °C. After purification with extraction buffer 2 (25 M sucrose, 10 mM Tris-HCl pH 8, 10 mM MgCl_2_, 1% Triton X-100, 5 mM BME) and extraction buffer 3 (1.7 M sucrose, 10 mM Tris-HCl pH 8, 2 mM MgCl_2_, 0.15% Triton X-100, 5 mM BME), the samples were washed with 1 ml of digestion buffer (320 mM sucrose, 50 mM Tris-HCl pH 8, 4 mM MgCl_2_, 1 mM CaCl_2_). 300 μl of digestion buffer was used to resuspend the pellet, and the samples were warmed up at 37 °C for 5 min. Then, 3 μl of MNase (Takara) was added to each sample, and the samples were incubated for 15 min at 37 °C. After digestion, 6 μl of 0.5 M EGTA (bioWORLD) and 6 μl of 0.5 M EDTA (Invitrogen) were added to quench the reaction at 65 °C for 10 min. Finally, 31.2 μl of 5 M NaCl (Invitrogen) was used to lyse the nuclei. DNA was recovered using the ChIP DNA Clean & Concentrator kit (Zymo Research) and run on a 2% gel to check the digestion efficiency. Next, the purified DNA was processed for the library using the Ovation Ultra Low System V2 kit (NuGEN). The sequencing was performed on an Illumina NovaSeq 6000.

### Flowering time measurement

Rosette and cauline leaves were counted to quantify the flowering time. Each dot in the dot plots (Fig. 1e and Extended Data Fig. 4a,b) represents the leaf count of a single plant. The cut-off line indicates plants with 22 leaves or fewer.

### Pollen extraction

Around 500 μl of open flowers were collected from six- to seven-week-old Col-0 and the related mutants into 2.0 ml Eppendorf tubes. Then, 700 μl of Galbraith buffer (45 mM MgCl_2_, 30 mM C_6_H_5_Na_3_O_7_·2H_2_O (trisodium citrate dihydrate), 20 mM MOPS, 0.1% (v/v) Triton X-100, pH 7) supplemented with 70 mM 2-mercaptoethanol was added, and the samples were vortexed at maximum speed for 3 min. The suspension containing mature pollen was filtered through an 80 μm nylon mesh (Component Supply) to a new 1.5 ml Eppendorf tube. Another 700 μl of Galbraith buffer was added to the flower samples, and the above procedure was repeated. The combined 1.4 ml of samples was centrifuged at 800 *g* at 4 °C, and the pollen pellet was collected. A metal bead was added to each sample for later grinding. The samples were flash-frozen in liquid nitrogen.

### RNA-seq

Biological triplicates were used for each genotype for RNA-seq. Mature pollen was harvested using the above protocol. One inflorescence containing unopened flower buds from a five- to six-week-old plant was collected as a biological replicate and frozen in liquid nitrogen. Rosette leaf tissues from four- to five-week-old plants were harvested from a single plant as a biological replicate and flash-frozen in liquid nitrogen. The samples were ground into powder, and RNA was extracted using the Direct-zol RNA MiniPrep kit (Zymo Research). 250 ng of total RNA from mature pollen and 1,000 ng of total RNA from inflorescences or leaf tissues were used for RNA-seq library preparation with the TruSeq Stranded mRNA kit (Illumina). The final library was sequenced on Illumina NovaSeq 6000 or HiSeq 4000 instruments.

### BS-PCR

Rosette leaf tissues from four- to five-week-old Col-0 and representative T_2_ MBD2–ZF108 transgenic lines that display the early flowering phenotype were collected for BS-PCR at the *FWA* promoter regions. DNA was extracted using the DNeasy Plant Mini kit (Qiagen). Around 2 μg of DNA was used for DNA bisulfate conversion with the EpiTect Bisulfate kit (QIAGEN). The converted DNA served as a template for amplification. PCR was performed at three different regions spanning the promoter and the 5′ transcribed regions of *FWA*, including region 1 (chr4: 13038143–13038272), region 2 (chr4: 13038356–13038499) and region 3 (chr4: 13038568–13038695). The PCR reactions used Pfu Turbo Cx (Agilent), dNTP (Takara Bio) and the primers designed for the above-mentioned *FWA* regions. PCR products from the same sample were pooled and cleaned using AMPure beads (Beckman Coulter). The purified PCR products were prepared for the libraries using the Kapa DNA Hyper Kit (Roche) with indexes from TruSeq DNA UD indexes for Illumina (Illumina). Finally, the libraries were sequenced on an Illumina iSeq 100.

### snRNA-seq

The snRNA-seq experiment was performed following the published protocol^29^. Each buffer was freshly added with 2-mercaptoethanol to reach 70 mM concentration and supplemented with cOmpleteTM Protease Inhibitor Cocktail (Sigma). In brief, 5 ml of unopened flower buds and open flowers from the same inflorescence were harvested on ice. A prechilled mortar and pestle were used to release the spores from the buds with 5 ml of 0.1 M mannitol. The liquid containing the released spores was transferred to a 50 ml conical tube. Another 10 ml of 0.1 M mannitol was used to rinse the mortar and pestle. The samples were then vortexed at maximum speed for 30 s to further release the spores. Next, the samples were filtered through a 100 μm nylon mesh (Component Supply) to remove the debris. Another 5 ml of 0.1 M mannitol was used to rinse the tubes and filtered through the same 100 μm nylon mesh. Then, 20 ml of the suspension containing the spores was filtered again through a 60 μm nylon mesh. The sample was distributed into two 15 ml glass tubes and centrifuged with a Sorvall Lynx 4000 Centrifuge (Thermo Scientific) with a TH13–6×50 swing-out rotor for 10 min at 900 *g* at 4 °C. Each pellet was resuspended in 1 ml of ice-cold 0.1 M mannitol and transferred into a new tube to layer over 3 ml of 20% Percoll. The samples were centrifuged again for 10 min at 450 *g* at 4 °C. The pellets from the same genotype were then combined with 2 ml of 0.1 M mannitol and centrifuged over 20% Percoll with the same settings two more times. The purified mixed spores were transferred into a 1.5 ml Eppendorf tube and centrifuged for 5 min at 500 *g* at 4 °C. 800 μl of Galbraith buffer was used to resuspend the pellet. The samples were then transferred to a 1.5 ml tube with 100 ml of acid-washed 0.5 mm glass beads (Sigma). To break the pollen cell walls, the samples were vortexed at maximum speed for 2 min at 4 °C with the following settings: for the first minute, 7 s vortex, 3 s inversion; for the second minute, 7 s vortex, 2 s inversion. A 10 mm cellTrics filter was placed in a clean 1.5 ml tube, and the suspension was added on and filtered by brief centrifugation. This flowthrough was kept on ice. The beads were rinsed again with 400 μl of Galbraith buffer, and the suspension was transferred to the cellTrics filter and centrifuged to collect more nuclei. Given that unbroken pollen grains remained on the filter, 800 μl of Galbraith buffer was added to transfer the suspension on the filter back into the tube with the glass beads. The vortexing and filtering were repeated. The combined suspension was then centrifuged for 5 min at 500 *g* at 4 °C, and the nuclei pellets were resuspended with 50 μl of CyStain UV Precise P—Nuclei Extraction Buffer (Sysmex, 05-5002-P02). 400 μl of CyStain UV Precise P—Staining Buffer (Sysmex, 05-5002-P01) was added to stain the nuclei, and the samples were added to Protector RNase Inhibitor (Sigma) to reach a final concentration of 0.2 U ml^−1^. The samples were passed into a FACS tube (Falcon 352235) and sorted immediately. Sorting was performed with a BD FACS ARIAII instrument equipped with a 355 nm UV laser, using the 70 mm nozzle. For each sample, 40,000–60,000 nuclei were sorted in 500 ml of nuclei wash buffer (2% BSA in 1× PBS) supplemented with Protector RNase Inhibitor (Sigma). The sorted nuclei were centrifuged for 5 min at 500 *g* at 4 °C. Finally, the pellet was resuspended in 20–25 μl of buffer and sent as input for the 10× Genomics Chromium Single Cell 30 Reagent Kit v.3.

### WGBS

For WGBS, rosette leaf tissues from four- to five-week-old Col-0 and representative T_2_ MBD2–ZF108 transgenic lines that display the early flowering phenotype were collected and frozen in liquid nitrogen. For mature pollen WGBS, 1,000 μl of open flowers were collected, and the pollen pellets were immediately frozen after purification. DNA from leaf tissues and mature pollen were extracted using the DNeasy Plant Mini kit (Qiagen). A total of 500 ng of DNA from leaf tissues and 100 ng of DNA from mature pollen was sheared to ~300 bp using the Covaris S2 (Covaris). The libraries were then constructed using the Ovation Ultralow Methyl-seq kit (NuGEN), and bisulfite conversion was achieved using the Epitect Bisulfite Conversion kit (QIAGEN). Finally, the libraries were sequenced on Illumina NovaSeq 6000 or HiSeq 4000 instruments.

### ChIP-seq analysis

For ChIP-seq analysis, quality control was initially run to filter out the low-quality reads. Trim Galore (v.0.6.7, Babraham Institute) was used to remove the Illumina adapters. The reads were then aligned to the *Arabidopsis* reference genome (TAIR10) using bowtie2 (v.2.3.4)^41^, allowing only uniquely mapped reads with perfect matches. MarkDuplicates.jar (picard-tools suite, v.3.1.0, Broad Institute) was used to remove the PCR duplicates. Samtools (v.1.9) was used to create indexes for the bam files. Bigwig files were generated using deeptools (v.3.0.2) bamCoverage^42^ with the options normalizeUsing RPGC and binSize 10. For correlation analysis between the ChIP-seq signal and mCG density, the samples were normalized to the no-FLAG control using deeptools (v.3.0.2) bamCompare^42^ with the options scaleFactorsMethod readCount, binSize 10 and operation log2. The normalized ChIP-seq signal and CG methylation percentages were summarized into 400 bp bins. We took a random subset covering 10% of all genomic regions for the correlation analysis. The data were plotted using the R package ggplot with the option geom_smooth. ChIP-seq peaks were called using MACS2 (v.2.1.1)^43^ using an FDR cutoff of 0.05. The FLAG-associated hyperchipable regions, defined as peaks called in the anti-FLAG Col-0 controls, were removed from the peak files. Heterochromatin peaks were defined as peaks intersecting with TAIR10 pericentromeric regions using the bedtools (v.2.30.0) intersect^44^ function from deeptools (v.3.0.2).

### MNase-seq analysis

MNase-seq reads of low quality were filtered out, and the adaptors were trimmed with Trim Galore (v.0.6.7, Babraham Institute). Next, the processed reads were aligned to TAIR10 using bowtie2 (v.2.3.4)^41^, keeping reads smaller than 2,000 bp and allowing only uniquely mapped reads with perfect matches. PCR duplicates were then removed using MarkDuplicate (picard-tools suite, v.3.1.0, Broad Institute), and bigwig files were generated using deeptools (v.3.0.2) bamCoverage^42^.

### RNA-seq analysis

RNA-seq reads were filtered according to quality score, and Illumina adaptors were trimmed out using Trim Galore (v.0.6.7, Babraham Institute). The filtered reads were then mapped to the *Arabidopsis* reference genome (TAIR10) using STAR (v.2.7.11a)^45^. We allowed only uniquely mapped reads with less than 5% mismatches. Bigwig files for genome browser visualization were generated using deeptools (v.3.0.2) bamCoverage^42^ with the options normalizeUsing RPGC and binSize 10. HTSeq (v.0.13.5)^46^ was used to obtain the read counts for TEs using our previously reannotated pollen transcripts, as described in the ‘Pollen transcriptome reannotation’ method section in ref. ^29^. DESeq2 (v.1.42.0) was used to perform the differential analysis with the cut-offs *P*_adj_ < 0.05 and |log_2_FC| ≥ 1 (to define whether a TE is activated or not, we used *P*_adj_ < 0.05 and log_2_FC ≥ 1). The number of activated TEs from the same genotype may vary due to the sequencing depth difference. For example, the number of *mbd2*-activated TEs is different between Figs. 2a and 3a. Data presented in box plots have been normalized to the Col-0 wild type. We used ggplot2 (v.3.4.4) to generate all the related plots. We took the union of the activated TEs from mutants to generate the box plots.

### snRNA-seq analysis

The snRNA-seq analysis was performed following the previously published pipeline^29^. In brief, Cell Ranger (v.6.1.1) was used to process the raw data following the published pollen transcriptome reannotations^29^. With the Cell Ranger results, SoupX (v.1.6.0)^47^ and Seurat (v.4.0.4)^48^ were used to remove the ambient RNA and filter out the cells detected with less than 200 genes. The data were normalized and scaled following the published settings^29^. After the normalization, PCA was performed (npc = 20). DoubletFinder (v.3.6)^49^ was used to identify doublets, and find.pK (DoubletFinder v.3.6) was used to obtain the ideal pK parameters for each sample. The percentage of doublets removed and the pK values are summarized in Supplementary Table 1. The Col-0 and *mbd2* datasets were integrated with Seurat (v.4.0.4) FindIntegrationAnchors and IntegrateData using the default settings. The data were scaled, and PCA was performed (npcs = 40). Clustering analysis was then done using the FindNeighbors and FindClusters functions in Seurat (v.4.0.4). The number of cells per cluster is summarized in Supplementary Table 6. In addition, the markers for each cluster were obtained with Seurat (v.4.0.4) FindAllMarker using the integrated dataset. Finally, DEG analysis was performed on individual clusters. We specifically focused on activated TEs using the cut-offs *P*_adj_ < 0.05 and |avg_log_2_FC| > 0.25. In this analysis, the following clusters were groups: VN_bi and VN_late_bi, VN_tri and VN_mature. The TE expression heat map was generated using the function AverageExpression in Seurat (v.4.0.4).

### WGBS analysis

WGBS reads were filtered and removed with Illumina adaptors using Trim Galore (v.0.6.7, Babraham Institute). Reads with three or more consecutively methylated CHH sites were considered as non-converted reads and removed from the analyses. Bismark (v.0.19.1, Babraham Institute)^50^ was used to map the reads to the *Arabidopsis* reference genome (TAIR10) and obtain the methylation percentages for each cytosine. We used ViewBS (v.0.1.11)^51^ to generate the plots showing the genome-wide methylation information across genotypes.

### Expression profile analysis

The expression profiles of MBD1, MBD2 and MBD4 were obtained from the Evorepro database (https://evorepro.sbs.ntu.edu.sg/) using Expression Heatmap (https://evorepro.sbs.ntu.edu.sg/heatmap/) (Supplementary Table 3). The expression level was row normalized.

### Reporting summary

Further information on research design is available in the Nature Portfolio Reporting Summary linked to this article.

### Supplementary information

Supplementary Information Supplementary Tables 1–6.

Reporting Summary

### Source data

Source Data Extended Data Fig. 4 Unprocessed western blots for Extended Data Fig. 4f.

## Data Availability

The high-throughput sequencing data generated in this paper have been deposited in the Gene Expression Omnibus database (accession no. GSE236290). The TAIR10 genome is available at https://www.arabidopsis.org/index.jsp. The expression profiles of MBD1, MBD2 and MBD4 were obtained from the Evorepro database (https://evorepro.sbs.ntu.edu.sg/). Source data are provided with this paper.

## References

[1] Greenberg MVC, Bourc’his D (2019). The diverse roles of DNA methylation in mammalian development and disease. Nat. Rev. Mol. Cell Biol..

[2] Smith ZD, Meissner A (2013). DNA methylation: roles in mammalian development. Nat. Rev. Genet..

[3] Law JA, Jacobsen SE (2010). Establishing, maintaining and modifying DNA methylation patterns in plants and animals. Nat. Rev. Genet..

[4] Zhang H, Lang Z, Zhu JK (2018). Dynamics and function of DNA methylation in plants. Nat. Rev. Mol. Cell Biol..

[5] Kankel MW (2003). *Arabidopsis*
*MET1* cytosine methyltransferase mutants. Genetics.

[6] Jones PL (1998). Methylated DNA and MeCP2 recruit histone deacetylase to repress transcription. Nat. Genet..

[7] Nan X (1998). Transcriptional repression by the Methyl-CpG-binding protein MeCP2 involves a histone deacetylase complex. Nature.

[8] Ichino L (2021). MBD5 and MBD6 couple DNA methylation to gene silencing through the J-domain protein SILENZIO. Science.

[9] Lai AY, Wade PA (2011). Cancer biology and NuRD: a multifaceted chromatin remodelling complex. Nat. Rev. Cancer.

[10] Denslow SA, Wade PA (2007). The human Mi-2/NuRD complex and gene regulation. Oncogene.

[11] Allen HF, Wade PA, Kutateladze TG (2013). The NuRD architecture. Cell. Mol. Life Sci..

[12] Zemach A, Grafi G (2003). Characterization of *Arabidopsis thaliana* Methyl-CpG-Binding Domain (MBD) proteins: Methyl-CpG-Binding Domain (MBD) proteins in *Arabidopsis*. Plant J..

[13] Scebba F (2003). *Arabidopsis* MBD proteins show different binding specificities and nuclear localization. Plant Mol. Biol..

[14] Ito M, Koike A, Koizumi N, Sano H (2003). Methylated DNA-binding proteins from *Arabidopsis*. Plant Physiol..

[15] Zemach A, Grafi G (2007). Methyl-CpG-Binding Domain proteins in plants: interpreters of DNA methylation. Trends Plant Sci..

[16] Wu Z (2022). Family-wide characterization of methylated DNA binding ability of *Arabidopsis* MBDs. J. Mol. Biol..

[17] Mahana Y (2022). Structural insights into methylated DNA recognition by the Methyl-CpG binding domain of MBD6 from *Arabidopsis thaliana*. ACS Omega.

[18] Zemach A (2005). DDM1 binds *Arabidopsis* Methyl-CpG Binding Domain proteins and affects their subnuclear localization. Plant Cell.

[19] Macek B (2019). Protein post-translational modifications in bacteria. Nat. Rev. Microbiol..

[20] Ytterberg AJ, Jensen ON (2010). Modification-specific proteomics in plant biology. J. Proteom..

[21] Zhang M, Xu JY, Hu H, Ye BC, Tan M (2018). Systematic proteomic analysis of protein methylation in prokaryotes and eukaryotes revealed distinct substrate specificity. Proteomics.

[22] Harris CJ (2018). A DNA methylation reader complex that enhances gene transcription. Science.

[23] Soppe WJJ (2000). The late flowering phenotype of Fwa mutants is caused by gain-of-function epigenetic alleles of a homeodomain gene. Mol. Cell.

[24] Johnson LM (2014). SRA- and SET-domain-containing proteins link RNA polymerase V occupancy to DNA methylation. Nature.

[25] Gallego-Bartolomé J (2019). Co-targeting RNA polymerases IV and V promotes efficient de novo DNA methylation in *Arabidopsis*. Cell.

[26] Guo, Y. et al. RAD: a web application to identify region associated differentially expressed genes. *Bioinformatics*10.1093/bioinformatics/btab075 (2021). 10.1093/bioinformatics/btab075 33532827

[27] Zhou X (2021). A domesticated *Harbinger* transposase forms a complex with HDA6 and promotes histone H3 deacetylation at genes but not TEs in *Arabidopsis*. J. Integr. Plant Biol..

[28] Feng C (2021). *Arabidopsis* RPD3-like histone deacetylases form multiple complexes involved in stress response. J. Genet. Genomics.

[29] Ichino L (2022). Single-nucleus RNA-seq reveals that MBD5, MBD6, and SILENZIO maintain silencing in the vegetative cell of developing pollen. Cell Rep..

[30] Borg M, Brownfield L, Twell D (2009). Male gametophyte development: a molecular perspective. J. Exp. Bot..

[31] Berger F, Twell D (2011). Germline specification and function in plants. Annu. Rev. Plant Biol..

[32] Calarco JP (2012). Reprogramming of DNA methylation in pollen guides epigenetic inheritance via small RNA. Cell.

[33] Borg M (2021). Epigenetic reprogramming rewires transcription during the alternation of generations in *Arabidopsis*. eLife.

[34] Zhao S (2019). Plant HP1 protein ADCP1 links multivalent h3k9 methylation readout to heterochromatin formation. Cell Res..

[35] Lee JH, Bollschweiler D, Schäfer T, Huber R (2021). Structural basis for the regulation of nucleosome recognition and HDAC activity by histone deacetylase assemblies. Sci. Adv..

[36] Sievers F (2011). Fast, scalable generation of high‐quality protein multiple sequence alignments using Clustal Omega. Mol. Syst. Biol..

[37] Goujon M (2010). A new bioinformatics analysis tools framework at EMBL-EBI. Nucleic Acids Res..

[38] McWilliam H (2013). Analysis tool web services from the EMBL-EBI. Nucleic Acids Res..

[39] Letunic I, Bork P (2021). Interactive Tree Of Life (ITOL) v5: an online tool for phylogenetic tree display and annotation. Nucleic Acids Res..

[40] Yan L (2015). High-efficiency genome editing in *Arabidopsis* using YAO promoter-driven CRISPR/Cas9 system. Mol. Plant.

[41] Langmead B, Salzberg SL (2012). Fast gapped-read alignment with Bowtie 2. Nat. Methods.

[42] Ramírez F (2016). DeepTools2: a next generation web server for deep-sequencing data analysis. Nucleic Acids Res..

[43] Zhang Y (2008). Model-Based Analysis of ChIP-Seq (MACS). Genome Biol..

[44] Quinlan AR, Hall IM (2010). BEDTools: a flexible suite of utilities for comparing genomic features. Bioinformatics.

[45] Dobin A (2013). STAR: ultrafast universal RNA-seq aligner. Bioinformatics.

[46] Anders S, Pyl PT, Huber W (2015). HTSeq—a Python framework to work with high-throughput sequencing data. Bioinformatics.

[47] Young MD, Behjati S (2020). SoupX removes ambient RNA contamination from droplet-based single-cell RNA sequencing data. GigaScience.

[48] Hao Y (2021). Integrated analysis of multimodal single-cell data. Cell.

[49] McGinnis CS, Murrow LM, Gartner ZJ (2019). DoubletFinder: doublet detection in single-cell RNA sequencing data using artificial nearest neighbors. Cell Syst..

[50] Krueger F, Andrews SR (2011). Bismark: a flexible aligner and methylation caller for bisulfite-seq applications. Bioinformatics.

[51] Huang X (2018). ViewBS: a powerful toolkit for visualization of high-throughput bisulfite sequencing data. Bioinformatics.

