# Supplementary material for: MBD2 couples DNA methylation to transposable element silencing during male gametogenesis
Source: Nat Plants. 2024 Jan 15;10(1):13–24. doi: 10.1038/s41477-023-01599-3 (PMC10808059; doi:10.1038/s41477-023-01599-3)
Supplement: Supplementary file 1 — Supplementary Tables 1–6. [file 41477_2023_1599_MOESM1_ESM.pdf]

nature plants

Letter

https://doi.org/10.1038/s41477-023-01599-3

MBD2 couples DNA methylation to transposable element silencing during male gametogenesis

In the format provided by the authors and unedited

Supplementary Table 1. DNA methylation information at MBD1/2/4 ChIP-seq peaks.

ChIPseq samples	total peak number	peaks with DNA methylation	% DNA methylated
MBD1	4227	500	12%
MBD2	16269	8772	54%
MBD4	9973	6963	70%

ChIPseq samples	peaks without DNA	% non-DNA methylated
MBD1	3727	88%
MBD2	7497	46%
MBD4	3010	30%

Supplementary Table 2. Classes/Families of TEs activated in diffent mutants.

	mbd2	*hda6*	*adcp1*	All TEs	% at heterochromatin
DNA/MuDR	30.0%	31.0%	38.0%	17.0%	51.2%
DNA	1.9%	1.5%	1.9%	5.9%	68.3%
LTR/Gypsy	26.0%	20.0%	19.0%	13.0%	90.4%
RC/Helitron	3.8%	7.5%	12.0%	42.0%	40.4%
DNA/Harbing	0.0%	1.5%	1.9%	1.2%	48.3%
DNA/En-Spm	11.0%	7.5%	1.9%	3.0%	75.0%
LTR/Copia	1.9%	13.0%	9.6%	5.7%	55.2%
LINE/L1	21.0%	13.0%	12.0%	4.4%	54.0%
Others	0.0%	1.5%	0.0%	7.4%	
Unassigned	4.0%	3.8%	3.8%	0.4%	
Total number	53	67	52	31189	

Color Scale
0%
25%
50%
75%
100%

Supplementary Table 3. Expression profile of MBD1/2/4 across tissues.

Gene	AT3G63030(MBD4)	AT4G22745(MBD1)	AT5G35330(MBD2)
Egg cell	0.21	0.06	0.24
Ovule	0.44	0.35	0.48
Uninucleate microspore (Col-0)	0.69	0.23	0.09
Uninucleate microspore (Ler-0)	0.86	0.35	0.18
Pollen (bicellular, Col-0)	0.55	0.25	0.32
Pollen (late bicellular, Col-0)	0.28	0.12	0.57
Pollen (bicellular, Ler-0)	1	0.32	0.33
Pollen (tricellular, Col-0)	0.05	0	0.23
Pollen (tricellular, Ler-0)	0.14	0	0.1
Pollen (mature, Col-0)	0.02	0	0.05
Pollen (mature, Ler-0)	0.07	0	0.06
Sperm	0.42	0	0.03
Pollen tube (Col-0)	0.02	0	0.07
Flower (receptacles)	0.47	0.22	0.39
Flower (floral buds)	0.09	0.24	0.18
Flower (sepals)	0.21	0.12	0.22
Flower (petals)	0.08	0.1	0.21
Flower (stamen filaments)	0.02	0.11	0.17
Flower (anthers)	0.11	0.13	0.1
Flower (carpels)	0.48	0.36	0.44
Flower (stigmatic tissue)	0.23	0.23	0.26
Pedicel	0.22	0.16	0.22
Axis of the inflorescence	0.38	0.22	0.3
Silique	0.24	0.16	0.24
Silique (senescent)	0.03	0.05	0.16
Pods of siliques	0.14	0.12	0.24
Pods of siliques (senescent)	0.13	0.1	0.23
Embryo	0.25	0.39	0.34
Endosperm	0.54	0.09	0.39
Seed	0	0.1	0.2
Seed (young)	0.36	0.37	0.26
Seed (germinating)	0.24	0.11	0.24
Seedling	0.07	0.1	0.22
Seedling (etiolated)	0.17	0.07	0.34
Meristem	0.82	0.4	0.38
Stem (internode)	0.27	0.11	0.32
Stem (internode, senescent)	0.36	0.2	0.72
Stems	0.43	0.16	0.4
Leaf (rosette)	0.18	0.12	1
Epidermis cells	0.01	0.02	0.09
Mature guard cells	0	0.04	0.04
Root (differentiation zone)	0.1	0.12	0.24
Root (elongation zone)	0.16	0.06	0.23
Root (meristematic zone)	0.14	0.1	0.34
Root (apex)	0.17	0.06	0.19
Root (stele)	0.03	0	0.56
Root (QC cells)	0.72	1	0.46
Root (tip)	0.14	0.05	0.18

(Values are normalized against highest expression of the row)

Color Scale

0
0.25
0.5
0.75
1

Supplementary Table 4. MBD2 sequences used for phylogenetic analysis.

Organism	Protein	Gene ID	Sequences used for phylogenetic analysis
Arabidopsis t	MBD1	AT4G22745	**PGLPRTPRGFKRSLILRKDYSKMDAYYITPTGKK LKSRNEIAAFIDANQDYKY**
Arabidopsis t	MBD2	AT5G35330	**PNISRPPAGWQRLLRIRGEGGTRFADVYYVAPSG KKLRSTVEVQKYLNDNSEYIG**
Arabidopsis t	MBD4	AT3G63030	**PGLPKTPKGFKRSLVLRKDYSKMDTYYFTPTGK KLRSRNEIAAFVEANPEFNA**
Arabidopsis t	MBD5	AT3G46580	**GDDNWLPPDWRTEIRVRTSGTKAGTVDKFYYEPI TGRKFRSKNEVLYYLEHGTPKKK**
Arabidopsis t	MBD6	AT5G59380	**PGDNWLPPGWRVEDKIRTSGATAGSVDKYYYEP NTGRKFRSRTEVLYYLEHGTSKRG**
Arabidopsis t	MBD7	AT5G59800	**SKGFRLPRGWSVEEVPRKNSHYIDKYYVERKTG KRFRSLVSVERYLRESRNSIE**
Arabidopsis t	MBD8	AT1G22310	**DYGGYLPRGWRLMLYIKRKGSNLLLACRRYISP DGQQFETCKEVSTYLRSLLESPS**
Arabidopsis t	MBD9	AT3G01460	**ERHGVLEDGWRVEFRQPLNGYQLCAVYCAPNG KTFSSIQEVACYLGLAINGNY**
Arabidopsis t	MBD10	AT1G15340	**SIELPAPASWKKLFYPKRAGTPRKTEIVFVAPTGE EISSRKQLEQYLKAHPGNPV**
Arabidopsis t	MBD11	AT3G15790	**SVELPAPSSWKKLFYPNKVGSVKKTEVVFVAPT GEEISNRKQLEQYLKSHPGNPA**
Homo sapien	MeCP2	P51608	**YDDPTLPEGWTRKLKQRKSGRSAGKYDVYLINP QGKAFRSKVELIAYFEKVGDTSL**

Supplementary Table 5. The information of the mutants used in this study.

Mutant	Description
mbd14	mbd1: SALK_025352 mbd4: SALK_042834
mbd124	mbd1: SALK_025352 mbd2: CRISPR (guides ACCGTAAATGCCCCGATAGA and CTAGGTACGCCAACCGAGTC) mbd4: SALK_042834
mbd56	mbd5: CRISPR (guides TCACGGAAACGTGCGACGCC and ACTTAGTATTTACTGATCGT) mbd6: SALK_043927
mbd256	mbd2: CRISPR (the same guides as above) mbd5: CRISPR (the same guides as above) mbd6: SALK_043927
mbd2 adcp1	mbd2: GABI_650A05 adcp1: CRISPR (guides ATTCCGCGGCTCGTGGTACATGG and GGCAGCTACCACTGAAAGGAGGG)

Supplementary Table 6. snRNA-seq summary of Col-0 and mbd2.

**Sample**	**Col0 exp1**	**mbd56 exp1**	
Estimated Number of Cells	15,689	10,869	
Mean Reads per Cell	16,340	20,639	
Median Genes per Cell	1,515	1,483	
Number of Reads	256,365,501	224,326,275	
Valid Barcodes	96.60%	97.10%	
Sequencing Saturation	54.50%	59.70%	
Q30 Bases in Barcode	95.30%	95.40%	
Q30 Bases in RNA Read	93.50%	92.90%	
Q30 Bases in UMI	94.80%	94.90%	
Reads Mapped to Genome	97.80%	97.80%	
Reads Mapped Confidently to Genome	82.80%	87.50%	
Reads Mapped Confidently to Intergenic Regions	3.20%	2.40%	
Reads Mapped Confidently to Intronic Regions	2.50%	2.30%	
Reads Mapped Confidently to Exonic Regions	77.00%	82.70%	
Reads Mapped Confidently to Transcriptome	69.40%	75.80%	
Reads Mapped Antisense to Gene	3.30%	2.60%	
Fraction Reads in Cells	78.00%	71.10%	
Total Genes Detected	32,422	31,061	
Median UMI Counts per Cell	2,959	2,962	
			**Total**
**sample**	**Col0 exp1**	**mbd2 exp1**	
tot n. cells after SoupX	15689	10869	
fraction of doublets	0.25	0.25	
pk	0.005	0.005	19919
n. cells after doublets removal	11767	8152	

**Number of nuclei per cluster**	**Col0exp1**	**mbd2exp1**
UNM	1876	1081
VN_bi	2209	1195
VN_late_bi	1724	1008
VN_tri	424	211
VN_mature	2510	1790
GN	1096	963
SN	1355	1608
Other	573	296

**Percentage of cells in each cluster (n. cells in cluster/total n. cells*100)**	**Col0exp1**	**mbd2exp1**
UNM	15.94289	13.26055
VN_bi	18.77284	14.65898
VN_late_bi	14.65114	12.36506
VN_tri	3.60330	2.58832
VN_mature	21.33084	21.95780
GN	9.31418	11.81305
SN	11.51525	19.72522
