# Supplementary material for: MBD2 couples DNA methylation to transposable element silencing during male gametogenesis
Source: Nat Plants. 2024 Jan 15;10(1):13–24. doi: 10.1038/s41477-023-01599-3 (PMC10808059; doi:10.1038/s41477-023-01599-3)
Supplement: Supplementary file 2 — Reporting Summary [file 41477_2023_1599_MOESM2_ESM.pdf]

nature portfolio

Corresponding author(s): Steven E. Jacobsen

Last updated by author(s): Nov 9, 2023

Reporting Summary

Nature Portfolio wishes to improve the reproducibility of the work that we publish. This form provides structure for consistency and transparency in reporting. For further information on Nature Portfolio policies, see our Editorial Policies and the Editorial Policy Checklist.

Statistics

For all statistical analyses, confirm that the following items are present in the figure legend, table legend, main text, or Methods section.

n/a	Confirmed	
☐	☒	The exact sample size (*n*) for each experimental group/condition, given as a discrete number and unit of measurement
☒	☐	A statement on whether measurements were taken from distinct samples or whether the same sample was measured repeatedly
☐	☒	The statistical test(s) used AND whether they are one- or two-sided<br>*Only common tests should be described solely by name; describe more complex techniques in the Methods section.*
☒	☐	A description of all covariates tested
☒	☐	A description of any assumptions or corrections, such as tests of normality and adjustment for multiple comparisons
☐	☒	A full description of the statistical parameters including central tendency (e.g. means) or other basic estimates (e.g. regression coefficient) AND variation (e.g. standard deviation) or associated estimates of uncertainty (e.g. confidence intervals)
☐	☒	For null hypothesis testing, the test statistic (e.g. *F*, *t*, *r*) with confidence intervals, effect sizes, degrees of freedom and *P* value noted<br>*Give P values as exact values whenever suitable.*
☒	☐	For Bayesian analysis, information on the choice of priors and Markov chain Monte Carlo settings
☒	☐	For hierarchical and complex designs, identification of the appropriate level for tests and full reporting of outcomes
☒	☐	Estimates of effect sizes (e.g. Cohen's *d*, Pearson's *r*), indicating how they were calculated

Our web collection on statistics for biologists contains articles on many of the points above.

Software and code

Policy information about availability of computer code

Data collection	No software was used for data collection.
Data analysis	Phylogenetic Analysis<br>Highly conserved MBD domain sequences of MBD1, MBD2, MBD4, MBD5, MBD6, MBD7, MBD8, MBD9, MBD10, MBD11, and human MeCP2 were taken for phylogenetic analysis. All the sequences were listed in Supplementary Table S2. Protein sequence alignments were performed using Clustal Omega. Graphic representation of the phylogenetic tree was generated using iTOL (v 6.7.5). Human MeCP2 was used as an outgroup given its evolutionary distance to Arabidopsis MBDs.<br><br>ChIP-seq analysis<br>Quality control was initially run to filter out the low-quality reads. Trim Galore (v 0.6.7, Babraham Institute) was used to remove the Illumina adapters. Then the reads were aligned to the Arabidopsis reference genome (TAIR10) using the bowtie2 (v 2.3.4), allowing only uniquely mapped reads with perfect matches. MarkDuplicates.jar (picard-tools suite, v 3.1.0, Broad Institute) was used to remove the PCR duplicates. Samtools (v 1.9) was used to create indexes for the bam files. Bigwig files were generated using deeptools (v 3.0.2) bamCoverage with the options -- normalizeUsing RPGC and --binSize 10. For correlation analysis between the ChIP-seq signal and mCG density, the samples were normalized to the no-FLAG control using deeptools (v 3.0.2) bamCompare with the options --scaleFactorsMethod readCount, --binSize 10, and --operation log2. The normalized ChIP-seq signal and CG methylation percentages were summarized into 400 bp bins. We took a random subset covering 10% of all genomic regions for the correlation analysis. The data were plotted using the R package ggplot with the option geom_smooth. ChIP-seq peaks were called using MACS2 (v2.1.1) using an FDR cutoff of 0.05. The FLAG-associated hyperchipable regions, defined as peaks called in the anti-FLAG Col-0 controls, were removed from the peak files. Heterochromatin peaks were defined as peaks intersecting with TAIR10 pericentromeric regions using bedtools (v 2.30.0) intersect function from deeptools (v 3.0.2).

MNase-seq analysis
The reads of low quality were fileterd out and the adaptors were trimmed with Trim Galore (v 0.6.7, Babraham Institute). Next the processed reads were aligned to TAIR10 using bowtie2 (v 2.3.4) keeping reads smaller than 2000 bp and allowing only uniquely mapped reads with perfect matches. Then PCR duplicates were removes using MarkDuplicate (picard-tools suite, v 3.1.0, Broad Institute) and bigwig files were generated using deeptools (v 3.0.2) bamCoverage.

RNA-seq analysis
RNAs-seq reads were filtered according to quality score and were trimmed out Illumina adaptors using Trim Galore (v 0.6.7, Babraham Institute). Then the filtered reads were mapped to the Arabidopsis reference genome (TAIR10) using STAR (v 2.7.11a). We allowed only uniquely mapped reads with less than 5% of mismatches. Bigwig files for genome browser visualization were generated using deeptools (v 3.0.2) bamCoverage with the options -- normalizeUsing RPGC and --binSize 10. HTSeq (v 0.13.5) was used to obtain the read counts for TE using our previously reannotated pollen transcripts, as described in the "Pollen transcriptome reannotation" method section in ref. DESeq2 (v 1.42.0) was used to perform the differential analysis with the cutoff padj < 0.05 and |log2FC| >= 1 (to define whether a TE is activated or not, we used padj < 0.05 and log2FC >=1). The number of activated TEs from the same genotype may vary due to the sequencing depth difference. For example, the number of mbd2-activated TEs is different between Fig.2a and Fig.3a. Data presented in boxplots has been normalized to Col-0 wild type. We used ggplot2 (v 3.4.4) to generate all the related plots. We took the union of the activated TEs from mutants to generate the boxplots.

Single-nuclei RNA-seq analysis
The analysis was performed following the published pipeline. In brief, Cell Ranger (v 6.1.1) was used to process the raw data following the published pollen transcriptome reannotations. With Cell Ranger results, SoupX (v 1.6.0) and Seurat (v 4.0.4) were used to remove the ambient RNA and filter out the cells detected with less than 200 genes. The data were normalized and scaled following the published settings. After the normalization, PCA analysis was performed (npc=20). DoubletFinder (v 3.6) was used to identify doublets and find.pK (DoubletFinder v 3.6) was used to obtain the ideal pK parameters for each sample. The percentage of doublets removed and the pK values were summarized in Table S1. Col-0 and mbd2 datasets were integrated with Seurat (v 4.0.4) FindIntegrationAnchors and IntegrateData using default settings. The data was scaled, and PCA analysis was performed (npcs=40). Then clustering analysis was done using Seurat (v 4.0.4) FindNeighbors and FindClusters functions. The number of cells per cluster is summarized in Table S1. In addition, the markers for each cluster were obtained with Seurat (v 4.0.4) FindAllMarker using the integrated dataset. Finally, DEG analysis was performed on individual clusters. We specifically focused on activated TEs using the cutoff padj < 0.05 & |avg_log2FC| > 0.25. In this analysis, the following clusters were groups: VN_bi and VN_late_bi, VN_tri and VAN_mature. The TE expression heatmap was generated using Seurat (v 4.0.4) function AverageExpression.

Whole-genome bisulfite sequencing analysis
WGBS were filtered and removed with Illumina adaptors using Trim Galore (v 0.6.7, Babraham Institute). Reads with three or more consecutively methylated CHH sites were considered as non-converted reads and removed from the analyses. Bismark (v 0.19.1, Barbraham Institute) was used to map the reads to the Arabidopsis reference genome (TAIR10) and obtain the methylation percentages for each cytosine. We used ViewBS (v 0.1.11) to generate the plots showing the genome-wide methylation information across genotypes.

Expression profile analysis
The expression profile of MBD1, MBD2, and MBD4 was obtained from Evorepro database (https://evorepro.sbs.ntu.edu.sg/) using Expression Heatmap (https://evorepro.sbs.ntu.edu.sg/heatmap/) (Supplementary Table S5). The expression level was row normalized.

For manuscripts utilizing custom algorithms or software that are central to the research but not yet described in published literature, software must be made available to editors and reviewers. We strongly encourage code deposition in a community repository (e.g. GitHub). See the Nature Portfolio guidelines for submitting code & software for further information.

Data

Policy information about availability of data

All manuscripts must include a data availability statement. This statement should provide the following information, where applicable:

- Accession codes, unique identifiers, or web links for publicly available datasets
- A description of any restrictions on data availability
- For clinical datasets or third party data, please ensure that the statement adheres to our policy

The high-throughput sequencing data generated in this paper have been deposited in the Gene Expression Omnibus (GEO) database (accession no. GSE236290, link: https://www.ncbi.nlm.nih.gov/geo/query/acc.cgi?acc=GSE236290). TAIR10 genome is available at https://www.arabidopsis.org/index.jsp. The expression profile of MBD1, MBD2, and MBD4 was obtained from Evorepro database (https://evorepro.sbs.ntu.edu.sg/).

Research involving human participants, their data, or biological material

Policy information about studies with human participants or human data. See also policy information about sex, gender (identity/presentation), and sexual orientation and race, ethnicity and racism.

Reporting on sex and gender	N.A.
Reporting on race, ethnicity, or other socially relevant groupings	N.A.
Population characteristics	N.A.
Recruitment	N.A.

Ethics oversight | N.A.

Note that full information on the approval of the study protocol must also be provided in the manuscript.

Field-specific reporting

Please select the one below that is the best fit for your research. If you are not sure, read the appropriate sections before making your selection.

☒ Life sciences ☐ Behavioural & social sciences ☐ Ecological, evolutionary & environmental sciences

For a reference copy of the document with all sections, see nature.com/documents/nr-reporting-summary-flat.pdf

Life sciences study design

All studies must disclose on these points even when the disclosure is negative.

Sample size	No sample size calculation was performed. Sample sizes are determined on experimental trials and a previous study (Wang et al., Nature Plants, 2023). Sample sizes of all experiments were large enough (e.g. unopen buds and open flowers from >=20 plants were collected for snRNA-seq; open flowers from >=20 plants were collected for bulk RNA-seq with three biological replicates; unopen buds from >=80 plants were harvested for ChIP-seq with two biological replicates and etc.) to reach statistical reproducibility and significance.
Data exclusions	No data exclusion in the study.
Replication	Two replicates for ChIP-seq. Two replicates for BS-PCR. Two replicates for WGBS. Three replicates for RNA-seq samples. Three technical replicates for qRT-PCR. All replicates were performed independently and produced high reproducible results.
Randomization	For all experiments, treatment and control samples were grown side by side, each replicate on separate plate. Allocation of samples were not random, because it is not relevant to the study.
Blinding	No blinding used because it was largely not relevant to our study. All data were collected based on the genotype of plants, while blinding the samples during the experiments will increases the risk of mislabeling and wrong results.

Reporting for specific materials, systems and methods

We require information from authors about some types of materials, experimental systems and methods used in many studies. Here, indicate whether each material, system or method listed is relevant to your study. If you are not sure if a list item applies to your research, read the appropriate section before selecting a response.

Materials & experimental systems

n/a	Involved in the study	
☐	☒	Antibodies
☒	☐	Eukaryotic cell lines
☒	☐	Palaeontology and archaeology
☒	☐	Animals and other organisms
☒	☐	Clinical data
☒	☐	Dual use research of concern
☒	☐	Plants

Methods

n/a	Involved in the study	
☐	☒	ChIP-seq
☒	☐	Flow cytometry
☒	☐	MRI-based neuroimaging

Antibodies

Antibodies used	Anti-FLAG Millipore Sigma Cat# F1804; RRID:AB_262044<br>Anti-FLAG M2-Peroxidase (HRP) Millipore Sigma Sigma-Aldrich Cat# A8592, RRID:AB_439702
Validation	Anti-FLAG M2 (Sigma): the antibodies have been validated by the manufacturer, https://www.sigmaaldrich.com/catalog/product/sigma/fl804<br>Anti-FLAG M2-Peroxidase (HRP)(Sigma): the antibodies have been validated by the manufacturer, https://www.sigmaaldrich.com/US/en/product/sigma/a8592

Plants

Seed stocks	Col-0 ecotype was obtained from SALK institute. T-DNA lines used in this study are listed as below: mbd1 (SALK_025352, from ABRC), mbd2 (GABI_650A05, from ABRC), mbd4 (SALK_042834, from ABRC), mbd6 (SALK_043927, from ABRC), hda6 (SALK_201895C, from ABRC), and sant3 (SALK_004966, from ABRC).
Novel plant genotypes	mbd2 CRISPR mutant was generated using guides: ACCGTAAATGCCCCGATAGA and CTAGGTACGCCAACCGAGTC. mbd5 CRISPR

Novel plant genotypes

mutant was generated using guides: TCACGGAAACGTGCGACGCC and ACTTAGTATTTACTGATCGT. adcp1 CRISPR mutant was generated using the same guides as Zhao, et al.: ATTCCGCGGCTCGTGGTACATGG and GGCAGCTACCACTGAAAGGAGGG. The sant1234 mutant is from Jian-Kang Zhu and Cui-Jun Zhang's group. Detailed information of high-order mutants generated in this study is summarized as below:
mbd14 mutant was generated by crossing mbd1 (SALK_025352) and mbd4 (SALK_042834)
mbd124 mutant was generated by crossing mbd1 (SALK_025352), mbd2 CRISPR mutant, and mbd4 (SALK_042834)
mbd56 mutant was generated by knocking out mbd5 via CRISPR-Cas9 in mbd6 (SALK_043927)
mbd256 mutant was generated by knocking out mbd2 and mbd5 via CRISPR-Cas9 in mbd6 (SALK_043927)
mbd2 adcp1 mutant was generated by knocking out adcp1 via CRISPR-Cas9 in mbd2 (GABI_650A05).

Authentication

T-DNA mutants were genotyped by PCR using the primers suggested by SALK (http://signal.salk.edu/tdnaprimers.2.html). CRISPR mutants were Sanger sequenced to confirm the mutations.

ChIP-seq

Data deposition

☒ Confirm that both raw and final processed data have been deposited in a public database such as GEO.

☒ Confirm that you have deposited or provided access to graph files (e.g. BED files) for the called peaks.

Data access links
May remain private before publication.

The high-throughput sequencing data generated in this paper have been deposited in the Gene Expression Omnibus (GEO) database (accession no. GSE236290, link: https://www.ncbi.nlm.nih.gov/geo/query/acc.cgi?acc=GSE236290).

Files in database submission

Col0-Flag-for-MBD2SANT3-rep1.bw
Col0-Flag-for-MBD2SANT3-rep2.bw
MBD2-139A-Flag-rep1.bw
MBD2-139A-Flag-rep2.bw
MBD2-160A-Flag-rep1.bw
MBD2-160A-Flag-rep2.bw
MBD2-Flag-rep1.bw
MBD2-Flag-rep2.bw
MBD2-RR-Flag-rep1.bw
MBD2-RR-Flag-rep2.bw
SANT3-Flag-rep1.bw
SANT3-Flag-rep2.bw
Col0-Flag-for-MBD4-Rep1.bw
Col0-Flag-for-MBD1-Rep2.bw
MBD1-Flag-Rep2.bw
MBD4-Flag-Rep1.bw
MBD1-Flag-rep1.bw
Col0-Flag-for-MBD1-rep1.bw
MBD4-104A-Flag.bw
MBD4-123A-Flag.bw
MBD4-FL-Flag.bw
MBD4-RR-Flag.bw
Col0-Flag-for-MBD4-rep2.bw
Col0-Flag-for-HDA6.bw
HDA6-Flag-Col0.bw
HDA6-Flag-mbd2CR.bw
HDA6-Flag-sant1234.bw
Col0-Flag-for-MBD2-sant3.bw
MBD2-Flag-sant3.bw
MBD2-139A-Flag-rep1_wohyperchip.narrowPeak
MBD2-139A-Flag-rep2_wohyperchip.narrowPeak
MBD2-160A-Flag-rep1_wohyperchip.narrowPeak
MBD2-160A-Flag-rep2_wohyperchip.narrowPeak
MBD2-Flag-rep1_wohyperchip.narrowPeak
MBD2-Flag-rep2_wohyperchip.narrowPeak
MBD2-RR-Flag-rep1_wohyperchip.narrowPeak
MBD2-RR-Flag-rep2_wohyperchip.narrowPeak
SANT3-Flag-rep1_wohyperchip.narrowPeak
SANT3-Flag-rep2_wohyperchip.narrowPeak
MBD1-Rep2_wohyperchip.narrowPeak
MBD4-Rep1_wohyperchip.narrowPeak
MBD1-rep1_wohyperchip.narrowPeak
MBD4-104A-Flag_B03_wohyperchip.narrowPeak
MBD4-123A-Flag_C03_wohyperchip.narrowPeak
MBD4-FL-Flag_A03_wohyperchip.narrowPeak
MBD4-RR-Flag_D03_wohyperchip.narrowPeak
HDA6-Flag-WT-rep2_wohyperchip.narrowPeak
HDA6-Flag-mbd2CR-rep2_wohyperchip.narrowPeak
HDA6-Flag-santnull-rep2_wohyperchip.narrowPeak
MBD2-Flag-sant3_wohyperchip.narrowPeak
MBD2-HDA6-shared_wohyperchip.narrowPeak

	Heterochromatin_methylated_MBD2_wohyperchip.narrowPeak<br>Heterochromatin_MBD4_wohyperchip.narrowPeak<br>HDA6-lostinsantnull-rep2_wohyperchip.narrowPeak<br>Random-Control_wohyperchip.narrowPeak
Genome browser session<br>(e.g. UCSC)	Available at GEO

Methodology

Replicates	2
Sequencing depth	Col0-Flag-rep1_S88_L003 48586708 43026870 150 PE<br>Col0-Flag-rep2_S82_L004 27514371 7436229 150 PE<br>Col0-Flag-rep2_S97_L003 40281056 34820118 150 PE<br>Col0-Flag_S6_L004 115299957 90341511 150 PE<br>Col0-Rep1_S49_L004 32641951 16853629 150 PE<br>Col0-Rep2_S50_L004 35862125 19335437 150 PE<br>HDA6-Flag-mbd2CR-rep2_S84_L004 18305136 7607612 150 PE<br>HDA6-Flag-santnull-rep2_S85_L004 19147727 9338577 150 PE<br>HDA6-Flag-WT-rep2_S83_L004 20556384 10117153 150 PE<br>MBD1-rep1_S54_L004 22953196 12677391 150 PE<br>MBD1-Rep2_S52_L004 96988681 71758505 150 PE<br>MBD2-139A-Flag-rep1_S89_L003 48348901 43090328 150 PE<br>MBD2-139A-Flag-rep2_S90_L003 44071143 39705185 150 PE<br>MBD2-160A-Flag-rep1_S91_L003 35196959 31441931 150 PE<br>MBD2-160A-Flag-rep2_S92_L003 65810298 59310829 150 PE<br>MBD2-Flag-rep1_S95_L003 57918146 49237299 150 PE<br>MBD2-Flag-rep2_S98_L003 50319515 38484472 150 PE<br>MBD2-Flag-sant3_S8_L004 102257088 73710505 150 PE<br>MBD2-RR-Flag-rep1_S93_L003 42738301 37628144 150 PE<br>MBD2-RR-Flag-rep2_S94_L003 54350626 50009461 150 PE<br>MBD4-104A-Flag_B03_merge 35920213 33766682 150 PE<br>MBD4-123A-Flag_C03_merge 30960057 28820720 150 PE<br>MBD4-FL-Flag_A03_merge 26025967 23212882 150 PE<br>MBD4-Rep1_S53_L004 75487698 61467880 150 PE<br>MBD4-RR-Flag_D03_merge 29780586 26826747 150 PE<br>SANT3-Flag-rep1_S96_L003 48098894 42018291 150 PE<br>SANT3-Flag-rep2_S99_L003 46795759 30930868 150 PE<br>WT-Flag_E03_merge 33102321 27912949 150 PE<br>WT-rep1_S45_L004 29546476 23502140 150 PE
Antibodies	Anti-FLAG M2 (Sigma)
Peak calling parameters	-g 1.3e+8 --bdg -q 0.05 -f BAM
Data quality	All identified peaks in the study were called with a qval threshold of 0.05 (FDR 5%).
Software	Trim Galore (v 0.6.7)<br>bowtie2 (v 2.3.4),<br>samtools (v 1.9)<br>MACS2 (v 2.1.1)<br>deeptools (v 3.0.2).<br>bedtools (v 2.30.0)<br>picard-tools suite (v 3.1.0)<br>STAR (v 2.7.11a)<br>HTSeq (v 0.13.5)<br>DESeq2 (v 1.42.0)<br>ggplot2 (v 3.4.4)<br>Cell Ranger (v 6.1.1)<br>Soup X (v 1.6.0)<br>Seurat (v 4.0.4)<br>DoubletFinder (v 3.6)<br>Bismark (v 0.19.1)<br>ViewBS (v 0.1.11)
