# Supplementary figures and images for: MBD2 couples DNA methylation to transposable element silencing during male gametogenesis
Source: Nat Plants. 2024 Jan 15;10(1):13–24. doi: 10.1038/s41477-023-01599-3 (PMC10808059; doi:10.1038/s41477-023-01599-3)

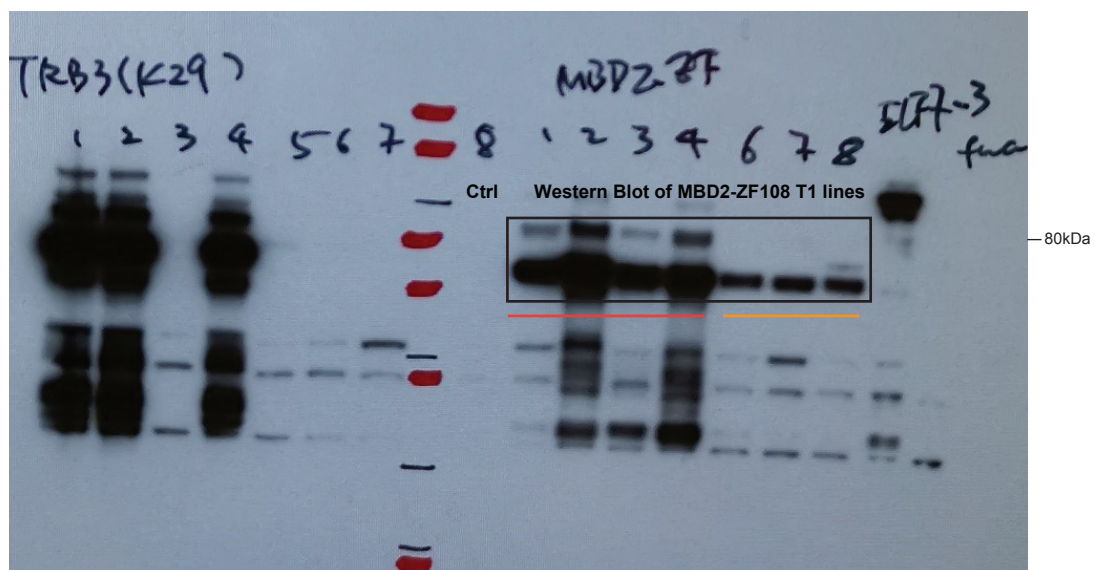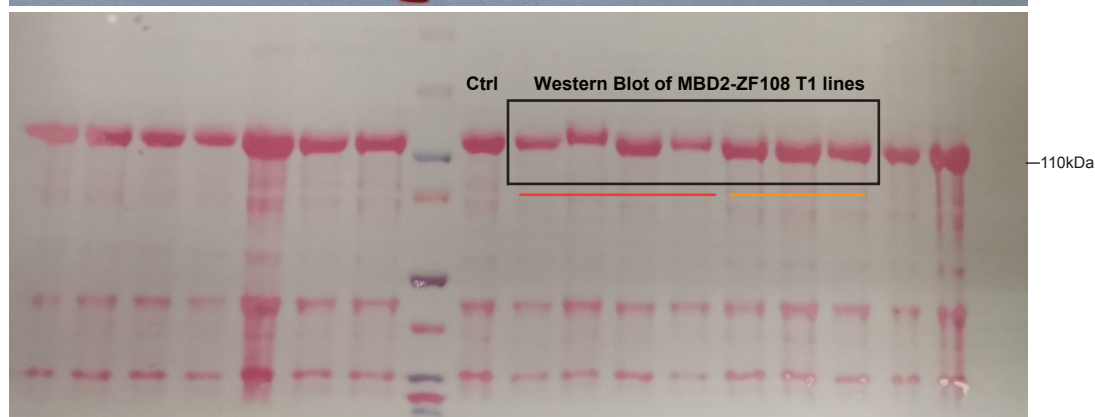

Supplement: Supplementary file 3 — Unprocessed western blots for Extended Data Fig. 4f. [file 41477_2023_1599_MOESM3_ESM.pdf]
